# DNA Methylation of Synaptic Genes in the Prefrontal Cortex Is Associated with Aging and Age-Related Cognitive Impairment

**DOI:** 10.3389/fnagi.2017.00249

**Published:** 2017-08-02

**Authors:** Lara Ianov, Alberto Riva, Ashok Kumar, Thomas C. Foster

**Affiliations:** ^1^Department of Neuroscience, McKnight Brain Institute, University of Florida, Gainesville FL, United States; ^2^Genetics and Genomics Program, Genetics Institute, University of Florida, Gainesville FL, United States; ^3^Bioinformatics Core, Interdisciplinary Center for Biotechnology Research, University of Florida, Gainesville FL, United States

**Keywords:** aging, cognitive flexibility, prefrontal cortex, set shifting task, epigenetics

## Abstract

The current study investigates DNA methylation as a possible epigenetic regulator of transcription associated with aging and cognitive function. Young and aged male Fischer 344 rats were behaviorally characterized on a set shifting task, and whole genome bisulfite sequencing was employed to profile the DNA methylome of the medial prefrontal cortex (mPFC). DNA methylation was also compared to RNA expression in the mPFC from the same animals. Variability in methylation was mainly observed for CpG sites as opposed to CHG and CHH sites. Gene bodies, specifically introns, contain the highest levels of methylation. During aging, hypermethylation was observed for genes linked to synaptic function and GTPase activity. Furthermore, impaired cognitive flexibility during aging was associated with hypermethylation of genes linked to postsynaptic density, dendrites, the axon terminus, and Ca^2+^ channels. Finally, comparison with RNA expression confirmed that hypermethylation was correlated with decreased expression of synaptic genes. The results indicate that DNA methylation over the lifespan contributes to synaptic modification observed in brain aging and age-related cognitive impairment.

## Introduction

Aging and age-related cognitive decline are associated with alterations in brain transcription linked to a number of functions including neuronal plasticity and synaptic wiring (Blalock et al., 2003; Lu et al., 2004; Stranahan et al., 2010; Uddin and Singh, 2013; Ianov et al., 2016b). The mechanism for altered transcription is unknown, but may involve epigenetic changes, including DNA methylation. DNA methylation is thought to play an important role in learning and memory (Day and Sweatt, 2011; Halder et al., 2016); however, due to technological limitations, much of the research on DNA methylation associated with brain aging and cognitive impairment has focused on methylation of CpG sites, particularly in genomic promoter regions of specific candidate genes in the hippocampus (Penner et al., 2011, 2016; Haberman et al., 2012; Ianov et al., 2016a). In contrast, genome-wide methylation studies, in several tissues, have revealed important DNA changes outside the promoter. For example, much of the DNA is comprised of repetitive elements, which can exhibit decreased methylation with age (Bollati et al., 2009; Jintaridth and Mutirangura, 2010; Talens et al., 2012). A decrease in methylation of these retrotransposable elements could result in genetic instability (Cordaux and Batzer, 2009; Li and Zhang, 2014). In addition, genome-wide methylation studies indicate an important role for methylation within the gene body and intergenic regions (Maunakea et al., 2010; Thompson et al., 2010; Guo et al., 2011; Oh et al., 2013; Zhang et al., 2017), which could influence transcription and alternative splicing (Jones, 2012). Finally, methylation of non-CpG sites may be of particular importance for regulating development and maturation of the brain (Xie et al., 2012; Lister et al., 2013; Guo et al., 2014; Sharma et al., 2016).

The medial prefrontal cortex (mPFC) is another brain region that is sensitive to aging and age-related cognitive decline. Previous profiling studies of the aging human prefrontal cortex have reported differential methylation of CpG sites related to brain development and the regulation of transcription (Hernandez et al., 2011; Numata et al., 2012); however, no study has investigated this epigenetic mark in the mPFC at the whole genome base pair resolution. Furthermore, the possible involvement of DNA methylation in aging of the mPFC and the decline in executive function remains to be elucidated. Recently, we reported that relative to young, the aging rodent mPFC is characterized by down regulation of synaptic, postsynaptic, and neuron projection genes, and up regulation of immune-related genes and oxidation-reduction genes (Ianov et al., 2016b). Furthermore, performance of aged animals on an executive function task was correlated with differential expression of genes associated with synaptic activity and regulation of transcription. The current study explores the DNA methylome in these same animals using whole genome bisulfite sequencing to investigate the impact of DNA methylation in aging and the mPFC’s executive function of cognitive flexibility.

## Materials and Methods

### Animals

Procedures involving animal subjects have been reviewed and approved by the Institutional Animal Care and Use Committee and were in accordance with guidelines established by the United States Public Health Service Policy on Humane Care and Use of Laboratory Animals. Male Fischer 344 rats of two ages, young (5–6 months, *n* = 10) and aged (17–22 months, *n* = 20) were obtained from National Institute on Aging colony (Taconic) through the University of Florida Animal Care and Service facility. Animals were maintained on a 12:12 h light schedule, and provided ad lib access to food and water prior to the set shifting task.

### Behavior and Gene Expression

The behavioral performance and differential gene expression for these animals have previously been reported (Ianov et al., 2016b). The initial study included eleven young animals. All but one young animal from the previous study were included in the current study examining DNA methylation. In this case, due to poor initial extraction of RNA from one of the mPFC, the contralateral side was required for RNA and could not be used for DNA methylation. For the current study, we focused on cognitive flexibility, using a set shifting task, which depends on the mPFC function. The methods for the set shifting task, tissue collection, and RNA-seq have been previously published (Ianov et al., 2016b). In brief, prior to the set shifting task, rats were reduced to 85% of their free feeding weights over the course of 5 days and maintained at this weight for the duration of the operant training and testing. Following behavioral shaping, animals were trained on visual discrimination, which required the animal to press the lever signaled by a light over the lever in order to obtain a reward. Upon the acquisition of visual discrimination, animals were tested in the set shifting phase, where the task parameters were changed such that the animal needed to press a lever based on location, and ignore the light, in order to obtain a reward. Our published behavioral data on these animals indicated no age difference in the ability of animals to acquire the visual discrimination (Ianov et al., 2016b). In contrast, an age difference was noted in the trials to criteria (TTC) on the set shifting task with young exhibiting fewer TTC relative to aged. However, aged animals exhibited substantial variability in their ability to shift their responding. In order to separate aged animals according to impairment, a mean split for the set shifting TTC score from the aged rats was performed to separate them into aged unimpaired rats (TTC < 51.7, *n* = 11) or aged impaired rats (TTC > 51.7, *n* = 9) and the behavioral characterization was used to identify changes in transcription associated with cognition (Ianov et al., 2016b). An ANOVA comparing TTC scores for young animals and aged animals characterized as impaired or unimpaired indicated group differences [*F*(2,28) = 23.84, *p* < 0.0001] and Fisher’s *post hoc* tests (*p* < 0.05) indicated that aged impaired (65.2 ± 4.2 mean ± SEM) exhibited more TTC than aged unimpaired (40.6 ± 2.2) and young (36.9 ± 2.8), which were not different from each other. Following completion of set shift testing, animals were returned to *ad libitum* food and water.

In order to minimize effects of behavioral testing, 2 weeks after completion of behavioral characterization, the mPFC (prelimbic and infralimbic regions) was collected and stored in -80°C until processed. Furthermore, the mRNA was enriched by poly-A selection, libraries were constructed, and sequencing was performed in the Ion Proton system (Thermo Fisher). The RNA-seq data is available in NCBI’s Gene Expression Omnibus under the accession number: GSE75772 and the results, relating to the mPFC RNA alterations in aging and cognitive function have been previously reported (Ianov et al., 2016b).

### Genomic DNA Isolation, Sodium Bisulfite Conversion and Library Preparation

Genomic DNA was isolated from the mPFC of the same animals in which the RNA-seq and behavioral data was previously published (Ianov et al., 2016b). Genomic DNA was isolated using the DNeasy Blood & Tissue kit (Qiagen, catalog number: 69504). The DNA concentration was quantified using the Qubit dsDNA HS Assay (Thermo Fisher, catalog number: Q32851) and sodium bisulfite conversion was performed with the EZ DNA Methylation-Direct kit (Zymo Research, catalog number: D5020) according to the manufacturer’s directions. Whole genome bisulfite sequencing (WGBS) libraries were constructed with the Illumina Truseq DNA Methylation kit (Illumina, catalog number: EGMK91324) with the following modifications: following terminal tagging of bisulfite converted DNA, purification was performed with SPRIselect reagent (Beckman Coulter, catalog number: B23317) for optimal size selection. Library size selection was completed using the SPRIselect reagent double-sided method, to remove fragments above and below the target size. The right size ratio used was 0.64 for a total of 32 μl of SPRIselect. The left side ratio used was 0.75, resulting in the ratio difference of 0.11 (left side minus right side ratios). Thus, 5.5 μl of SPRIselect was used for the second selection. Following size selection, amplification of the WGBS libraries was performed with a total of 17 cycles and with the addition of a unique barcode per library for multiplex sequencing with the TruSeq DNA Methylation Index PCR Primers (Illumina, catalog number: EGIDX81312). Successful amplification of each library was visualized with the 2% agarose SizeSelect E-Gel (Thermo Fisher, catalog number: G661002). Finally, libraries were purified using the Agencourt AMPure XP beads (BeckMan Coulter, catalog number: A63880) following the Truseq DNA Methylation kit directions. The concentration of the libraries was quantified by the Qubit dsDNA HS Assay and size distribution was evaluated with the High Sensitivity D1000 Screen Tape in the Tapestation system (Agilent Technologies).

### Sequencing, Bioinformatics, and Statistical Analysis

Paired-end sequencing of the WGBS libraries was performed with an Illumina NextSeq 500 (2x 101 bp) at the University of Florida Interdisciplinary Center for Biotechnology Research core. Multiplex sequencing of WGBS libraries was performed with RNA-seq libraries from a collaborator (50:50 ratio from each library type) to introduce base diversity to each sequencing cycle. Furthermore, 1% of PhiX spike-in control was added to improve the generation of base calls. On average, each biological sample contained a total of 133 million paired-end reads.

The data analysis was performed using the differential methylation analysis pipeline (DMAP2) available at the University of Florida high performance computer (HPC) clusters (Riva, 2016b). In short, DMAP2 is a new pipeline based on the MOABS pipeline (Sun et al., 2014), but with a number of improvements including better quality control and the ability to more accurately control methylation calls for each biological replicates. The steps from DMAP2, which were used for the current study include: (1) read trimming and quality control, which were performed with trimmomatic and FastQC (Andrews, 2010; Bolger et al., 2014). (2) Bisulfite conversion filtering of unconverted bases which was executed by the ‘cscall’ program (Riva, 2016a). (3) Alignment to the rn5 genome using BSMAP (Xi and Li, 2009). (4) DNA methylation calling at CpG sites which was performed with the ‘cscall’ program. The methylation calling parameters were set such that each site detected per group contained an effective coverage of least 15× (minimum coverage per site = 5; minimum number of animals per group = 3). Following the specified parameters, the average genome-wide coverage per CpG site was: 75.4 (young), 73.4 (aged), 74.4 (aged-unimpaired), and 70.1 (aged-impaired). In addition, all replicates contained bisulfite conversion rates above 95%. Furthermore, in order to assess variability among biological replicates, Pearson’s correlation was performed across all CpGs in all biological replicates in the aged and young groups. This analysis shows that the range for the *r*-values among the young animals was from 0.75 to 0.78. Aged animals contained r values from 0.75 to 0.80. Further, the methylated sites were annotated with the ‘genediffmeth’ program available at the University of Florida HPC. Annotation was performed using the Rnor_5.0.78.gtf file, and all sites were annotated according to promoters and gene body (exons and introns) regions.

To address the abundance level of non-CG methylation, the DNA methylation calling step was also performed at CHG and CHH sites (where H represents non-G bases: A, T, or C) with ‘cscall’ at the same coverage levels as CpG. The genome-wide coverage for the detected CHG sites were: 60.7 (young), 65.3 (aged), 61.1 (aged-unimpaired), and 57.5 (aged-impaired). The genome-wide coverage for the detected CHH sites were: 61.7 (young), 67.7 (aged), 62.4 (aged-unimpaired), and 58.9 (aged-impaired). The data for the current study, including the FASTQ files and the DNA methylation ratios for all sites in CG, CHG, and CHH contexts, have been uploaded to NCBI’s Gene Expression Omnibus under the accession number: GSE97612.

Following DNA methylation call and annotation, DNA methylation values from each CpG site and non-CG site were extracted for each biological replicate and statistical comparisons were made for aging and for age impairment. Statistical filtering was employed to obtain gene lists for cluster analysis (Blalock et al., 2003; Aenlle et al., 2009; Ianov et al., 2016b). For differential methylation analysis associated with age, Welch *t*-tests were performed between the aged and young, and a *p*-value of <0.05 was applied as a statistical filter. For the analysis of differential methylation associated with cognitive flexibility impairment, Pearson’s correlations were calculated between TTC scores for the set shifting task, and the DNA methylation ratios for each site. Correlations were limited to the aged animals in order to remove age as a confound. Pearson’s correlation values corresponding to a *p*-value < 0.05 (*r* = 0.444) were used as a statistical filter for the analysis.

For gene enrichment and functional annotation clustering analysis, data sets of hypomethylated or hypermethylated genes were separately submitted to the NIH database for annotation, visualization, and integrated discovery (DAVID, version 6.8) (Huang et al., 2007a,b). The cutoff for cluster selection from DAVID was set to the Benjamini False Discovery Rate (FDR) *p* < 0.05 and the ‘Direct’ and ‘FAT’ categories were used for gene ontology (GO) annotation. The circular genome map figures were generated by Circos using the rn5 genome ideogram obtained from the University of California, Santa Cruz (UCSC) genome browser (Karolchik et al., 2004; Krzywinski et al., 2009). Repetitive elements, identified by RepeatMasker, and CpG island locations were also acquired from the UCSC genome browser for the rn5 genome. The box plots were generated in R with ggplot2 (2.2.1).

## Results

### DNA Methylome Profiling at CpGs of the Aging mPFC

**FIGURE 1:**
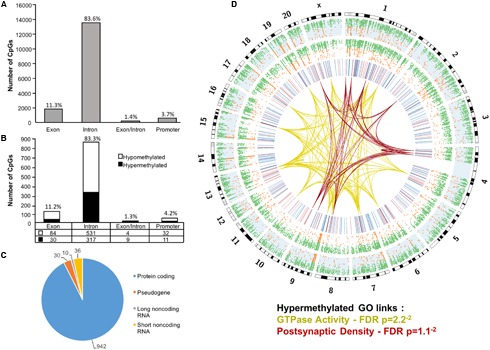
Genomic distribution of CpG sites in the aging mPFC. **(A)** Total CpG sites detected across young and aged rats and annotated according to genomic location in exons, introns, exon/intron boundaries, and promoters. **(B)** Differentially methylated sites (*p* < 0.05) whose methylation increased (filled) or decreased (open) with aging. A large number of CpG sites were located in the gene body regions relative to the promoter. **(C)** The diagram represents the relative proportion of CpGs in the gene body and promoter regions, which were differentially methylated during aging, and associated with protein coding genes (blue), short non-coding RNA (yellow), pseudogenes (orange), and long non-coding RNA (gray). The numbers indicate the number of CpG sites for each gene type. **(D)** Circos plot displaying the relationship of the DNA methylome profile to functional gene clusters for the aging mPFC. The outer track displays the rat genome ideogram by chromosome number. The second track indicates the distribution of all detected CpGs in the aged rats (blue background, *n* = 20) followed by the distribution in young in the third track (*n* = 10). Green dots are CpGs with methylation rates >50%; orange dots are CpGs with methylation rates <50%. The fourth track is a heatmap of differentially methylated CpGs (*p* < 0.05) relative to aged rats (hypermethylated – red; hypomethylated – blue). The links represent GO clusters for GTPase activity (yellow) and postsynaptic density (red) for CpGs, which were hypermethylated in aged animals. The legend indicates the FDR corrected p-value for each cluster.

Across the age groups, a total of 16,090 CpG sites were detected in promoter and gene body regions (average sequencing depths in promoter and gene body regions: young – 63.8, and aged – 130.7). The majority of identified sites (94.9%) were found in the gene body regions (**Figure 1A**). Among these sites, a statistical filter for age differences in DNA methylation (*p* < 0.05) was applied, which resulted in a total of 367 hypermethylated CpGs, and 651 hypomethylated CpGs in aged rats (FDR = 0.75). The distribution of the differentially methylated CpGs was characterized according to genomic location, which indicated that the majority of the differentially methylated CpGs were in introns (83.3%), followed by exons (11.2%), promoters (4.2%), and exon/intron boundaries (1.3%) (**Figure 1B**). In addition, 942 differentially methylated CpG sites were annotated to protein coding genes (592 hypomethylated, 350 hypermethylated), followed by 10 long non-coding (4 hypomethylated, 6 hypermethylated), 36 short non-coding (all hypomethylated) and 30 pseudogenes (19 hypomethylated, 11 hypermethylated) (**Figure 1C**).

**FIGURE 2:**
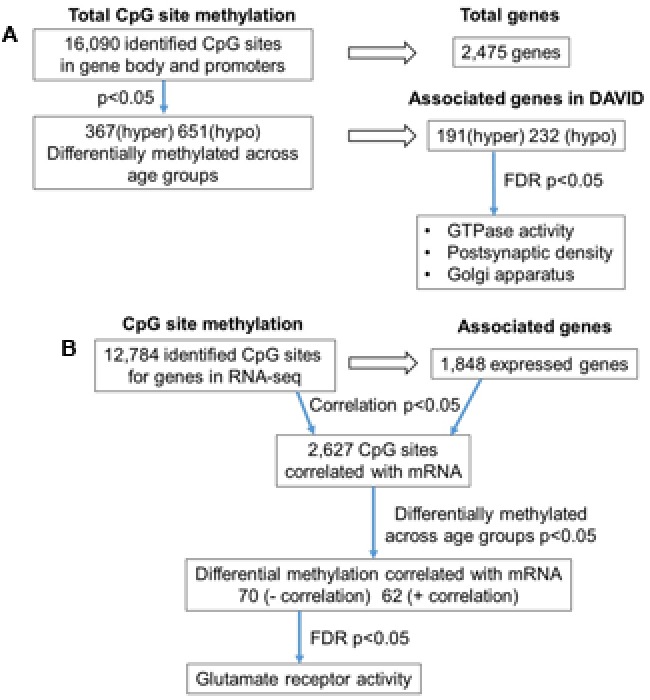
Diagrams summarizing age-related differences in CpG methylation. **(A)** Summary of total sites detected, differentially methylated sites, and associated gene clusters in the mPFC. The blue arrows indicate the analysis steps where a statistical cutoff was applied to determine differentially methylated sites (*p* < 0.05) in aged rats relative to young, and enrichment of genes containing differentially methylated CpG sites (FDR *p* < 0.05). **(B)** Summary of the relationship of CpG methylation and RNA expression in the aging mPFC. The blue arrows indicate the analysis steps where a statistical cutoff was applied to determine DNA to RNA correlation (Pearson’s correlation *p* < 0.05), differential methylation in aged rats relative to young (*p* < 0.05) and enrichment analysis (FDR *p* < 0.05).

The 16,090 CpG sites detected in promoter and gene body regions, corresponded to 2,475 genes and hyper- or hypomethylation during aging was associated with 424 genes annotated in DAVID. In order to determine functional clustering associated with differential methylation, the corresponding 191 hypermethylated and 232 hypomethylated genes were separately submitted to NIH DAVID for enrichment analysis of GO terms. Overall, age-related changes in the DNA methylome were primarily associated with hypermethylation of genes linked to GTPase activity (GO:0043087, 17 genes, FDR *p* = 2.2^-2^) and postsynaptic density (GO:0014069, 11 genes, FDR *p* = 1.1^-2^) (**Figure 1D**) (Supplementary Table 1). Furthermore, enrichment for the cellular component GO cluster for the golgi apparatus was also observed (GO:0005794, 25 genes, FDR *p* = 1.5^-2^). Enrichment analysis did not indicate significant clusters for hypomethylated genes. A summary diagram of the results from CpG site detection to GO analysis is shown in **Figure 2A**.

To investigate the relationship between DNA methylation and transcription associated with aging, the WGBS dataset was compared to previously published RNA-seq data from the same animals (Ianov et al., 2016b). A summary diagram of the correlation analysis is shown in **Figure 2B**. The comparison between the datasets identified 12,784 CpG sites from promoter and gene body regions, which corresponded to 1,848 genes, which were found in the RNA-seq dataset. Pearson’s correlation identified 2,627 CpGs in which DNA methylation for a gene was correlated to RNA expression (*p* < 0.05, *r* = 0.361), regardless of age (FDR = 0.24). When the analysis was limited to the 1,018 CpG sites, which were different with age, 70 differentially methylated CpGs were negatively correlated to RNA expression (Supplementary Table 2). Likewise, 62 differentially methylated CpGs across the age groups were positively correlated to RNA expression (Supplementary Table 2). Among the age-relevant CpGs that negatively correlated with RNA expression, a single significant cluster was observed, glutamate receptor activity (GO:0008066, 3 genes, FDR *p* = 1.3^-2^), which contained the following genes with increased methylation during aging: *Grik2*, *Grm5*, *Grm1*. It is also interesting to note that *Ppp1r9a*, which is linked to postsynaptic function, was observed to exhibit increased methylation that correlated with decreased expression (Supplementary Table 1).

**FIGURE 3:**
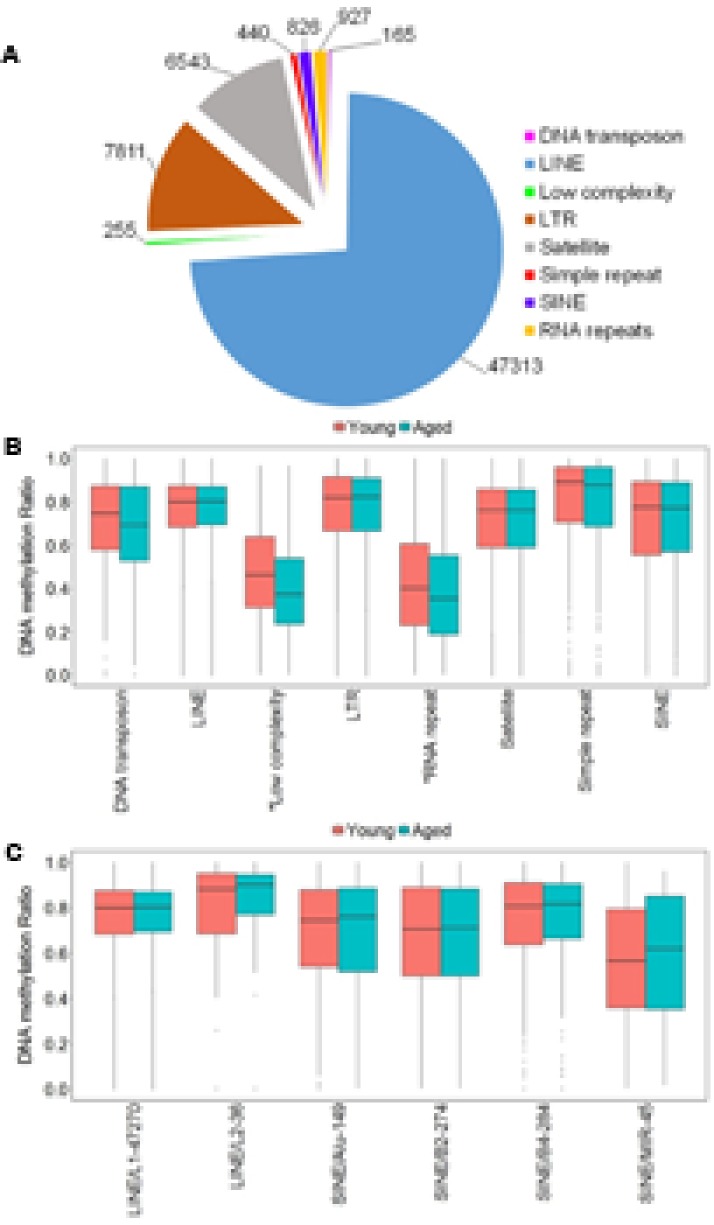
DNA methylation of repetitive elements in the aging mPFC. **(A)** Total number of detected CpG sites within repetitive elements across the genome. **(B)** Genome-wide DNA methylation ratios across the repetitive elements in young and aged rats. The gray dots indicate the outliers from the boxplots. The asterisks indicate a significant difference for age observed across all CpG sites within low complexity and RNA repeats (*p* < 0.05). **(C)** DNA methylation from the LINE and SINE families. The *x*-axis indicates the class, family, and number of CpG sites within each family, respectively. The gray dots indicate outliers within each family. The genome-wide methylation levels of the indicated families were not different between young and aged rats.

Due to the high occurrence of CpGs in repetitive elements across mammalian genomes, we investigated the abundance of the DNA methylation in repetitive elements of the rat mPFC for the aging dataset (Cordaux and Batzer, 2009; Su et al., 2012; Darby et al., 2016). The genomic locations from repetitive elements were downloaded from the UCSC genome browser identified by RepeatMasker for the rn5 genome which contained several classes of repetitive elements including DNA transposons, long interspersed nuclear elements (LINE), long terminal repeats (LTR), short interspersed nuclear elements (SINE), low complexity repeat, simple repeat, satellites, and RNA repeat. The locations of the repetitive elements were intersected to the chromosomal location of all CpG sites in promoters, exons, introns and all other sites outside of intragenic regions of young and aged animals. The total number of CpG sites within repetitive elements was 64,280, where the top three classes containing the most sites were: LINE (47,313 CpGs), followed by LTR (7,811 CpGs), and satellite (6,543 CpGs) (**Figure 3A**). Furthermore, the genome-wide DNA methylation ratios between young and aged rats were similar among most repeat types; however, there was a significant difference for age in low complexity repeats and RNA repeats (*p* < 0.05), with decreased methylation ratios for aged animals (**Figure 3B**).

In other tissues, DNA methylation of specific families from the LINE and SINE classes have been associated with diseases, including cancer (Igarashi et al., 2010; Richardson et al., 2015). Therefore, we quantified DNA methylation in the most abundant families from LINE and SINE. The L1 family from the LINE group contained the highest number of CpGs (47,270) while the L2 family contained 36 CpGs. The SINE class was subdivided into Alu, B2, B4, and MIR families, which contained 149, 274, 284, and 45 CpGs, respectively (**Figure 3C**). However, *t*-tests of the genome-wide methylation levels among the elements were not different with aging (**Figure 3C**).

Next, we investigated the abundance of repetitive elements in the 1,018 CpG sites, which were located in the promoter and gene body regions and were differentially methylated across age groups. Relative to young, aged rats exhibited 235 hypermethylated CpGs (8 in exons, 221 in introns, 2 in exons/introns, and 6 in promoter regions) and 418 hypomethylated CpGs within repetitive elements (32 in exons, 376 in introns, 1 in exon/intron, and 9 in promoter regions). Hypomethylated and hypermethylated genes were separately submitted to DAVID for each class of repetitive elements; however, no significant clustering was observed.

Lastly, CpG sites were annotated to regions belonging to CpG islands downloaded from the UCSC browser. Across the entire genome, we identified 2,511 CpGs, which belong to 146 islands. Among these, only a small number of sites within promoter and gene bodies were differentially methylated across the age groups: 39 hypomethylated CpGs were in islands, and 12 hypermethylated sites belonged to islands. In addition, among the differentially methylated sites, no overlap between sites in CpG islands and repetitive elements were observed.

### mPFC DNA Methylome Profiling at CpGs in Relation to Cognitive Flexibility

**FIGURE 4:**
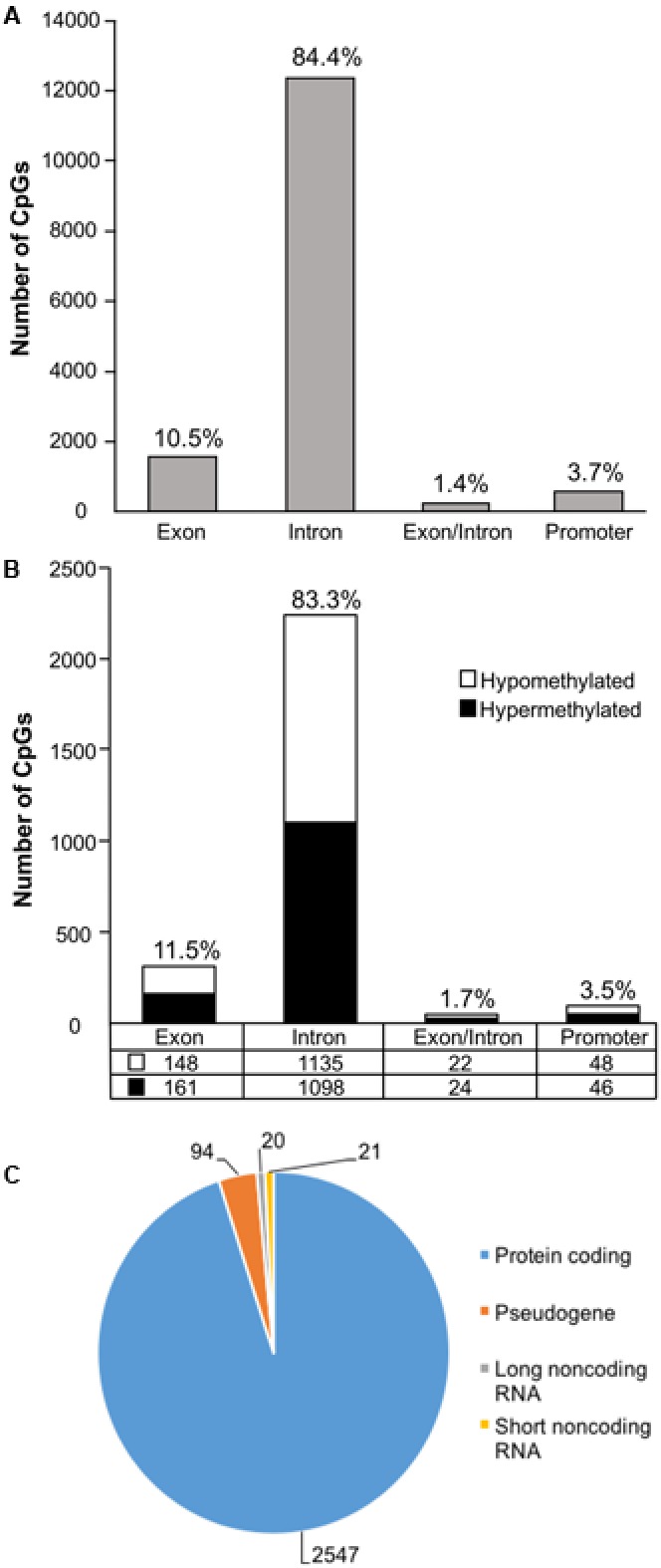
Genomic distribution of CpG sites relative to cognitive flexibility performance in aged rats. **(A)** Total CpG sites detected across all aged rats were annotated according to genomic location in exons, introns, exon/intron boundaries, and promoters. **(B)** Differentially methylated sites (Pearson’s correlation, *p* < 0.05) whose methylation increased (filled) or decreased (open) in aged-impaired rats. A large number of CpG sites were located in the gene body regions relative to the promoter. **(C)** The diagram represents the relative proportion of CpGs in the gene body and promoter regions, which were differentially methylated according to cognitive function, and associated with in protein coding genes (blue), short non-coding RNA (yellow), pseudogenes (orange), and long non-coding RNA (gray). The numbers indicate the number of CpG sites for each gene type.

In order to examine DNA methylation related to cognitive function, sites were called separately for aged animals characterized as impaired (AI) or unimpaired (AU). Similar to the aging analysis, CpG calls were limited to sites that exhibited a minimum of 15× coverage per group (i.e., at least three animals in each group). The total number of CpG sites detected across the age impaired and unimpaired rats in the promoter and gene body regions was 14,696, which corresponded to 2,123 genes (average sequencing depths in promoter and gene body regions: aged unimpaired – 77.6, and aged impaired – 67.3). Similar to the aging dataset, the majority of the sites detected (94.9%) were found in the gene body regions (**Figure 4A**). Among these sites, Pearson’s correlation analysis was performed between the set shifting TTC scores and methylation for each CpG site to investigate the relationship between DNA methylation changes and the ability to shift responses (*p* < 0.05, *r* = 0.444). The results indicated 2,682 sites in which methylation correlated with behavior (FDR = 0.27). For these sites, 1,329 CpGs were positively correlated, such that increased methylation was associated with delayed shifting, and 1,353 CpGs were negatively correlated with delayed shifting. The genomic distribution of CpGs, correlated to delayed shifting, was similar to that observed for age differences, with the majority of the sites being present in introns (83.3%), followed by exons (11.5%), promoters (3.5%), and exon/intron boundaries (1.7%) (**Figure 4B**). In addition, 2,547 differentially methylated CpG sites were annotated to protein coding genes (1,285 hypomethylated, 1,262 hypermethylated), 20 long non-coding RNA genes (11 hypomethylated, 9 hypermethylated), 21 short non-coding RNA genes (8 hypomethylated, 13 hypermethylated) and 94 pseudogenes (49 hypomethylated, 45 hypermethylated) (**Figure 4C**).

**FIGURE 5:**
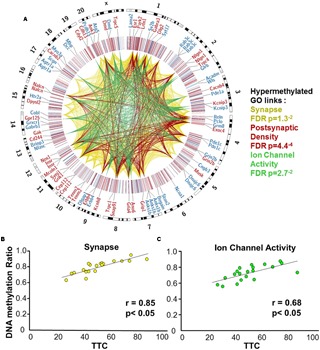
DNA methylation patterns associated with delayed set shift behavior in aged rats. **(A)** The outer circle of the Circos plot highlights a subset of genes from CpGs, which were correlated (hypermethylated – red, hypomethylated – blue) with behavior for animals characterized as aged-impaired (*n* = 9) and aged-unimpaired (*n* = 11), followed by a heatmap of all significant CpGs (Pearson’s correlation *p* < 0.05). The links represent CpG sites associated with genes containing GO clusters for synapse (yellow), postsynaptic density (red), and ion channel activity (green). The legend indicates the FDR corrected *p*-value for each cluster. **(B,C)** Pearson’s correlation between TTC scores of each aged animal (represented by each circle) and the average DNA methylation rates of all CpG sites from the gene clusters related to **(B)** the synapse (40 genes; 60 CpG sites) and **(C)** ion channel activity (25 genes; 35 CpG sites).

**FIGURE 6:**
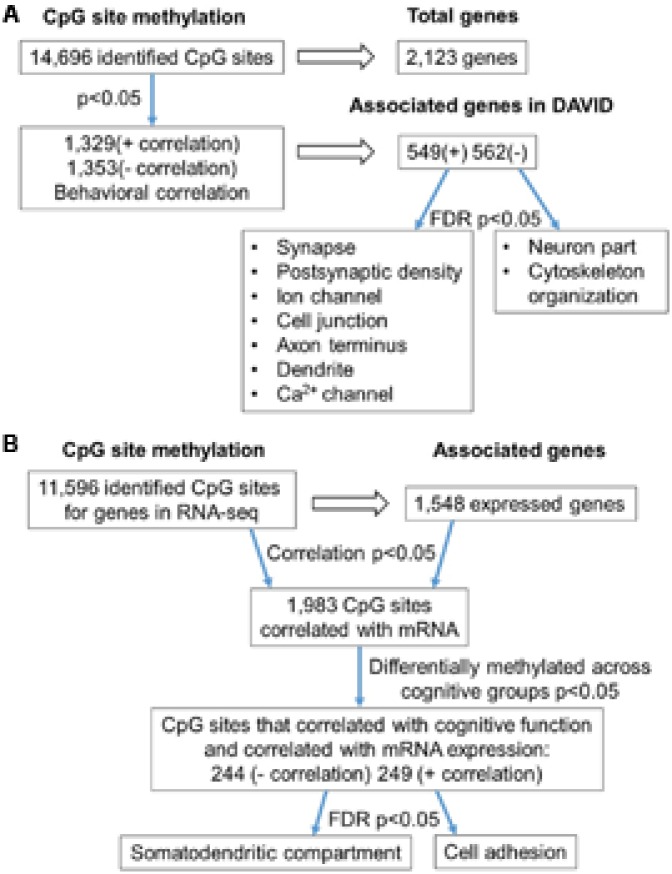
Diagram summarizing CpG methylation in aged-impaired and aged-unimpaired rats. **(A)** Summary of total sites detected, differentially methylated, and associated to gene clusters according to set shifting performance. The blue arrows indicate the analysis steps where a statistical cutoff was applied to determine differentially methylated sites (Pearson’s correlation, *p* < 0.05) in aged-impaired relative to aged-unimpaired. Finally, functional categories were revealed using enrichment analysis (FDR *p* < 0.05) of genes for methylated CpG sites that positively (+) or negatively (–) correlated with the TTC score. **(B)** Summary of the correlation of CpG methylation to RNA expression in aged-impaired and aged-unimpaired rats. The blue arrows indicate the analysis steps where a statistical cutoff was applied as a criterion for further analysis. The first cutoff required a correlation of DNA methylation and RNA expression (Pearson’s correlation *p* < 0.05). Next the methylation sites had to exhibit a correlation with behavior (Pearson’s correlation *p* < 0.05). Finally, enrichment analysis was performed (FDR *p* < 0.05) which resulted in a subset of genes which exhibited negative (–) or positive (+) correlation functionally related to the GO terms indicated.

Gene enrichment analysis was performed to identify biological function related to differential methylation correlated with set shifting behavior. For the 1,329 CpGs that were positively correlated with delayed shifting (i.e., hypermethylated in impaired animals), 549 genes were annotated in DAVID, and for the 1,353 CpGs which were negatively correlated with delayed shifting (i.e., hypomethylated in impaired animals), 562 genes were annotated in DAVID. Hypermethylation was observed for genes linked to synapse (GO:0045202, 40 genes, FDR *p* = 1.3^-2^), postsynaptic density (GO:0014069, 21 genes, FDR *p* = 4.4^-4^), and ion channel activity (GO:0005216, 25 genes, FDR *p* = 2.7^-2^) (**Figures 5A–C**) (Supplementary Table 3). Additional clusters linked to cell junction (GO:0030054, 25 genes, FDR *p* = 7.9^-3^), axon terminus (GO:0043679, 14 genes, FDR *p* = 3.8^-2^), dendrite (GO:0030425, 31 genes, FDR *p* = 2.1^-2^), and calcium channel activity (GO:0005262, 12 genes, FDR *p* = 1.9^-2^) were also observed. Hypomethylation in delayed shifting was correlated to cellular component clusters linked to neuron part (GO:0097458, 70 genes, FDR *p* = 3.4^-2^), and cytoskeleton organization (GO:0007010, 51 genes, FDR *p* = 4.7^-2^) (Supplementary Table 4). A summary diagram of the results from CpG site detection among aged animals to GO analysis is shown in **Figure 6A**.

Next, the results were compared to the RNA-seq dataset to examine the relationship of DNA methylation and RNA levels among the aged individuals belonging to impaired and unimpaired groups. Considering all CpG sites in the promoter and gene body regions in aged animals, across cognitive groups, 11,596 CpGs corresponded to 1,548 genes in the RNA-seq data. Pearson’s correlation across all aged-impaired and aged-unimpaired rats resulted 1,983 CpGs which were correlated to RNA levels (*p* < 0.05, *r* = 0.444) (FDR = 0.29). However, in order to focus on set shifting performance, a statistical filter was employed, such that only CpGs sites, which exhibited a correlation between DNA methylation and TTC scores (*p* < 0.05, *r* = 0.444), were retained for subsequent correlation with mRNA expression and gene enrichment analysis. Among the sites that were correlated with set shifting performance, 244 CpGs were negatively correlated to mRNA (Supplementary Table 5). Likewise, 249 CpG sites were positively correlated with mRNA (Supplementary Table 5). Cluster analysis for genes that negatively correlated with CpG methylation indicated enrichment of RNA involved in the cellular component of the somatodendritic compartment (GO:0036477, 20 genes, FDR *p* = 2.8^-2^). For genes that positively correlated with CpG methylation, enrichment was observed for cell adhesion (GO:0007155, 27 genes, FDR *p* = 4.4^-2^). However, many of the genes have also been linked to regulation of synaptic contacts (*Cntn4*, *Kirrel, Nfas, Dscaml1, Ctnna2, Cntnap5b, Cntnap5c, Efna5, Il1rapl1, Nlgn1, Phldb2, Ptk2*). A summary diagram of the correlation analysis is shown in **Figure 6B**.

### Non-CG Methylation in the mPFC

**FIGURE 7:**
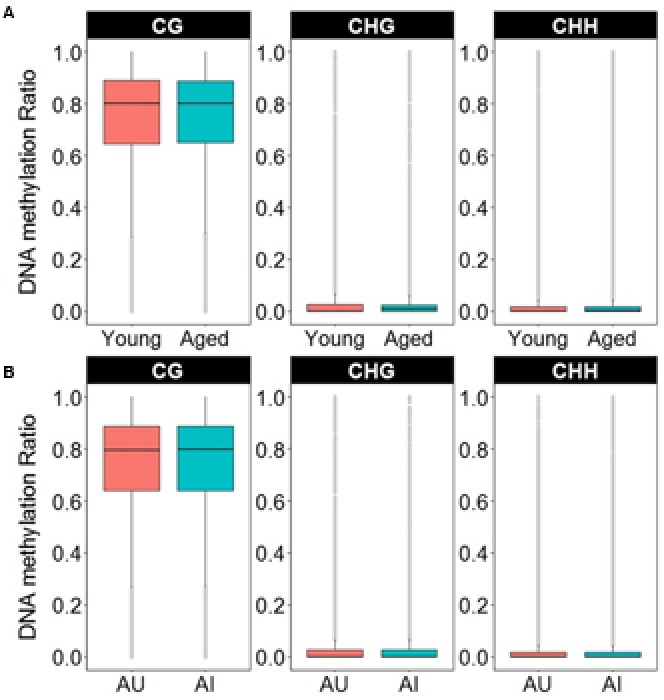
Boxplots of DNA methylation from the gene body and promoters in CpG, CHG, and CHH sites according to **(A)** age or **(B)** cognitive function. The gray dots indicate the outliers for each boxplot. Note that in contrast to CpG sites, the majority of non-CpG sites contained DNA methylation levels less than 10%.

In order to access the abundance of non-CG methylation in promoters and gene body regions of the mPFC, CHG, and CHH sites were quantified in young and aged animals. Supplementary Table 6 shows the total number of sites detected and the DNA methylation ratio for each context analyzed. While more sites were detected in non-CG context, the majority of the non-CG sites detected contained DNA methylation levels of less than 10%, with an overall average of less than 2.5% across all age groups (**Figure 7A** and Supplementary Table 6). In contrast, for CpG sites, only 3.5% of the sites in young, and 3.6% of the sites in aged animals contained ratios of less than 10%, with an overall average of 73% methylation across all sites in the promoter and gene body (**Figure 7A** and Supplementary Table 6). Thus, to reduce the chance of false positives, non-CG sites that contained methylation levels of less than 10% in both age groups were filtered out from our analysis.

### Non-CG Methylation and Aging

#### CHG Sites

**FIGURE 8:**
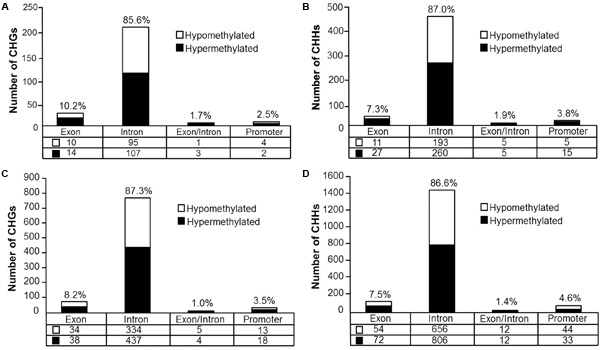
Genomic distribution of CHG and CHH sites according to aging **(A,B)** or cognitive flexibility performance **(C,D)**. **(A,B)** Differentially methylated sites (*p* < 0.05) whose methylation increased (filled) or decreased (open) in aged rats relative to young at **(A)** CHG sites and **(B)** CHH sites. **(C,D)** Genomic distribution of CHG and CHH sites relative to cognitive flexibility performance in aged rats. Differentially methylated sites (Pearson’s correlation, *p* < 0.05) whose methylation increased (filled) or decreased (open) in aged-impaired rats relative to aged-unimpaired at **(C)** CHG sites and **(D)** CHH sites.

Analysis revealed 126 hypermethylated (*p* < 0.05) (corresponding to 74 genes) and 110 hypomethylated sites (corresponding to 78 genes) CHG sites in aged animals (**Figure 8A**). Gene enrichment analysis was performed to identify biological function related to differential methylation of CHG sites in aging. Among the hypomethylated genes, 59 genes were annotated in DAVID, and clustering was observed for GTPase activity (GO:0043087, 9 genes, FDR *p* = 1.2^-2^). Among the hypermethylated genes, 47 genes were annotated in DAVID, however, clustering did not pass our FDR cutoff.

The relationship between methylation of CHG sites and RNA levels was examined, regardless of age. Overall, in the CHG dataset, 2,413 sites detected corresponded to 750 genes in the RNA-seq dataset. Pearson’s correlation across all rats resulted in 526 sites which were correlated to RNA levels (*p* < 0.05, *r* = 0.361). For the 526 sites in which methylation correlated with RNA expression, only 65 sites exhibited differential methylation across age groups, with 20 differentially methylated sites negatively correlated to RNA levels, and 45 differentially methylated sites positively correlated to RNA levels. Enrichment analysis did not indicate significant clusters for RNA associated with differentially methylated CHG sites.

#### CHH Sites

For CHH sites that exhibited methylation greater than 10% in at least one of the age groups, 307 sites were hypermethylated (*p* < 0.05, corresponding to 138 genes) and 214 sites were hypomethylated (corresponding to 110 genes) in aged rats (**Figure 8B**). Gene enrichment analysis of differentially methylated CHH sites showed that among the hypomethylated sites, 76 genes were annotated in DAVID; however, clustering did not pass our FDR cutoff. Furthermore, among the hypermethylated CHH sites, 98 genes were annotated in DAVID, but the gene list did not significantly cluster.

Examination of all CHH sites that exhibited methylation greater than 10%, regardless of age, indicated a total of 5,061 sites, which corresponded to 1,045 genes from the RNA-seq dataset. Among these sites, Pearson’s correlation was performed across all young and aged rats which resulted in 1,116 sites correlated to RNA levels (*p* < 0.05, *r* = 0.361). Among the CHH sites which correlated to RNA expression, 47 CHH sites, which were negatively correlated to RNA, were also differentially methylated across age groups (*p* < 0.05) and 35 CHH sites, which were positively correlated to RNA, were differentially methylated during aging. Enrichment analysis did not indicate any significant clusters.

### Non-CG Methylation and Cognitive Flexibility

#### CHG Sites

DNA methylation in non-CG context was also quantified for aged animals according to performance on the set shifting task. The average levels of DNA methylation in non-CG relative to CpG were similar to the aging dataset, where over 95% of the non-CG sites contained methylation ratios of less than 10% (Supplementary Table 7 and **Figure 7B**). Therefore, to reduce the chance of false positives, sites that contained methylation levels of less than 10% in both cognitive performance groups were filtered from our analysis. Thus, for the CHG context, 497 sites (corresponding to 276 genes) were positively correlated to TTC scores while 386 sites (corresponding to 228 genes) were negatively correlated to set shift behavior (**Figure 8C**). Gene lists of negatively and positively correlated genes were submitted to DAVID which resulted in 176 negatively correlated genes (hypomethylated for animals that delayed shifting) and 219 positively correlated genes (hypermethylated for animals that delayed shifting) annotated in the database. Among the hypermethylated CHG sites, GOs linked to kinase activity (GO:0016301, 22 genes, FDR *p* = 5.9^-3^), regulation of GTPase activity (GO:0043087, 19 genes, FDR *p* = 5.8^-3^) and synapse (GO:0045202, 23 genes, FDR *p* = 4.9^-3^) were observed (Supplementary Table 8). Clustering did not pass our FDR cutoff among the hypomethylated genes.

The results were compared to the RNA-seq dataset to examine the association of DNA methylation on non-CG context to RNA levels among the aged animals. Overall, in the CHG dataset, 2,467 sites detected in aged animals, which corresponded to 671 genes in the RNA-seq dataset. Pearson’s correlation across all aged rats resulted 494 sites which were correlated to RNA levels (*p* < 0.05, *r* = 0.444). Among the sites which correlated to RNA, 81 CHG sites which negatively correlated to RNA were also correlated to TTC scores, and 97 CHG sites which were positively correlated to RNA were also correlated to TTC scores. Enrichment analysis did not indicate significant clusters for RNA associated with differentially methylated CHG sites.

#### CHH Sites

For DNA methylation at CHH sites, 766 CHH sites (corresponding to 364 genes) were negatively correlated to the TTC score (high methylation in animals that readily shifted), and 923 CHH sites (corresponding to 397 genes) were positively correlated in animals that delayed shifting (**Figure 8D**). The lists of CHH hypomethylated and CHH hypermethylated genes were separately submitted to DAVID, which resulted in 300 hypomethylated genes and 322 hypermethylated genes annotated in DAVID. Among the hypermethylated genes, a single cluster passed our FDR cutoff: adenyl ribonucleotide binding (GO:0032559, 39 genes, FDR *p* = 2.3^-2^). Clustering did not pass our FDR cutoff for clusters of hypomethylated genes.

Finally, analysis was performed to examine the relationship between CHH site methylation and RNA expression. The total number of possible CHH sites was 5,163, which corresponded to 946 genes from the RNA-seq dataset. Among these sites, Pearson’s correlation was performed across the aged rats which resulted in 1,011 CHH sites correlated to RNA expression levels (*p* < 0.05, *r* = 0.444). Among the sites which correlated to RNA expression, 184 CHH sites which negatively correlated with RNA were also correlated to the TTC scores, and 184 CHH sites which were positively correlated with RNA were also correlated to the TTC scores. No significant clusters were observed following enrichment analysis.

## Discussion

Transcriptional studies indicate that several biological processes, including synaptic function, are altered during aging and associated with cognitive decline (Blalock et al., 2003; Verbitsky et al., 2004; Rowe et al., 2007; Ianov et al., 2016b). Furthermore, DNA methylation has been linked to synaptic plasticity and memory suppressor genes in the hippocampus, suggesting that DNA methylation has a role in cognitive function (Levenson et al., 2006; Miller and Sweatt, 2007; Day and Sweatt, 2010; Feng et al., 2010; Lardenoije et al., 2015). However, previous research on DNA methylation associated with brain aging and cognitive impairment has focused on methylation of CpG sites, particularly in genomic promoter regions of specific candidate genes in the hippocampus (Penner et al., 2011, 2016; Haberman et al., 2012; Ianov et al., 2016a). The development of techniques for genome-wide analysis of DNA methylation provides the opportunity to test multiple hypotheses concerning the relationship between DNA methylation and cognitive decline during aging (Liu et al., 2009). The current study presents several novel findings that relate to the idea that DNA methylation contributes to differential gene expression associated with aging and linked to cognitive function.

The results presented in the current study touch upon the general and specific nature of DNA methylation. First, the direction of differential methylation, hypermethylation vs. hypomethylation, was not specific for age or cognitive function, as approximately half the differentially methylated sites were hypermethylated. The results indicate that the pattern of methylation is not due to a general mechanism such as increased expression or activity of a methyltransferase. Second, compared to CHG and CHH sites, CpG sites are much more likely to be methylated, indicating some specificity. In contrast, CpG methylation does not appear to be specific for genomic location. The distribution of differential methylation across genomic location associated with aging or cognitive function was similar to the distribution of available sites with the majority of DNA methylation located within gene body regions, particularly introns.

DNA methylation at promoter regions has been well studied as a mechanism for regulation of transcription (Moore et al., 2013). In addition, methylation of CpG sites transcriptionally silence transposons to prevent genetic instability (Cordaux and Batzer, 2009; Li and Zhang, 2014). In contrast, the function of gene body methylation in regulating transcription is debated. While a number of studies have reported that gene body methylation is positively associated with transcriptional activity (Hellman and Chess, 2007; Wu et al., 2010; Jones, 2012; Yang et al., 2014), several studies have also indicated repression of expression, or no clear pattern, due to evidence for the activation and repression of genes in the same dataset (Guo et al., 2011; Jjingo et al., 2012; Oh et al., 2013; Debski et al., 2016; Sharma et al., 2016; Neri et al., 2017). A recent study proposed that *Dnmt3b* dependent gene body methylation is associated with the repression of aberrant transcription in embryonic stem cells (Neri et al., 2017). While the expression of *Dnmt3b* is lower in the brain, *Dnmt1* and *Dnmt3a* are highly expressed in postmitotic neurons and are fundamental to memory and synaptic plasticity, raising the possibility that they may have a role in the mechanism of gene body methylation (Feng et al., 2010; Moore et al., 2013). One study described a relationship of gene body methylation and gene expression, which followed a bell-shaped distribution, such that low and highly expressed genes contained the least amount of methylation, with mid-level genes containing the highest levels of DNA methylation (Jjingo et al., 2012). The current work supports the hypothesis that gene body methylation may have more than one regulatory mechanism since several differentially methylated CpGs for age or cognitive function were positively and negatively correlated to RNA levels.

In contrast to an absence in specificity for the overall direction of methylation, specificity was observed in terms of the genes that exhibited differential methylation. Aging was associated with hypermethylation of genes linked to synaptic function and GTPase activity, processes that have been linked to age-related cognitive decline and diseases of aging (Pavlidis et al., 2004; Erraji-Benchekroun et al., 2005; Bishop et al., 2010; Bossers et al., 2010; Twine et al., 2011; Berchtold et al., 2013). An important finding of the current study was that cognitive impairments in aged animals on a task that depends on the mPFC was associated with DNA methylation in this brain region. Again, hypermethylation was observed for genes linked to the postsynaptic density, dendrites, the axon terminus, and Ca^2+^ channels. In the case of animal studies that permit examination of the molecular basis of age-related cognitive decline, research points to altered Ca^2+^ signaling and a decrease in expression of RNA for synaptic components in impaired animals (Blalock et al., 2003; Stranahan et al., 2010; Uddin and Singh, 2013; Schafer et al., 2015; Ianov et al., 2016b).

The specificity of gene changes supports the idea that the DNA may be dynamically modified across the life span, resulting in alterations to gene expression, which influences synaptic connectivity and cognition (Levenson et al., 2006; Xu, 2015; Ianov et al., 2016a). Alternatively, the specificity may be due to ongoing transcription, which renders these sites available for methylation or demethylation. Thus, an important question is whether the methylation we observed influenced transcription and is linked to brain aging and cognitive decline. Determining the relationship of DNA methylation and the pattern of RNA expression may be masked by the heterogeneity of cell types. Previous studies have distinguished different methylation patterns for glial cells and neurons (Lister et al., 2013). Furthermore, it is likely that gene expression depends on a dynamic interaction of epigenetic regulation with transcriptional signals such as neural activity leading to activation of transcription factors. Finally, while we provide a 2 weeks delay between behavioral testing and examination of DNA methylation, it is possible that experience produces long-lasting changes in DNA methylation (Miller et al., 2010; Provencal et al., 2012; Mychasiuk et al., 2013; Anier et al., 2014; Baker-Andresen et al., 2015; Barbier et al., 2015). Despite these possible confounds, we found that hypermethylation during aging or in cognitively impaired animals was correlated with decreased expression of genes linked to synaptic function. The decrease in gene expression is consistent with work in the dentate gyrus showing hypermethylation of CpG and CHG sites is associated with decreased gene expression (Guo et al., 2014). Specifically, hypermethylation during aging was correlated with decreased expression of three glutamate receptor genes (*Grik2, Grm5*, and *Grm1*), and *Ppp1r9a*, which localizes protein phosphatase 1 to the synapse. Similarly, hypermethylation in cognitively impaired animals was associated with decreased expression of the *N*-methyl-D-aspartate receptor subunit, *Grin2b* and *Nlgn1*, which is involved in localizing proteins to the synapse, and *Stau2*, an RNA-binding protein required for transport of neuronal RNA from the cell body to the dendrite. The results are consistent with the idea that DNA methylation over the lifespan contributes to synaptic modification observed in brain aging and cognitive impairments in aged animals.

Considerable work across species indicate that aging is consistently associated with an increased expression of immune response genes across different brain regions (VanGuilder et al., 2011; Cribbs et al., 2012; Ianov et al., 2016b; Hargis and Blalock, 2017) and a decrease in expression of RNA for synaptic genes (Blalock et al., 2003; Lu et al., 2004; Loerch et al., 2008; Berchtold et al., 2013; Ianov et al., 2016b). However, when examined, age-related cognitive decline is correlated with the decrease in synaptic genes, rather than the increase in immune genes. Furthermore, the relationship between cognitive function and differential expression of genes linked to synaptic activity and plasticity is specific for brain regions that underlie the cognitive process examined (Ianov et al., 2016b). Thus, it will be interesting to determine if genome-wide analysis of DNA methylation in the hippocampus would reveal similar epigenetic mechanisms for synaptic genes in aged animals that are impaired on hippocampal-dependent tasks.

In summary, the current study profiled the genome-wide DNA methylation changes associated with aging and cognitive impairment in the mPFC at single base pair resolution. The results suggest that while the alterations in the number of hypomethylated and hypermethylated CpG sites are similar across aging and cognitive performance, hypermethylation is more likely to be associated with decreased expression of genes linked to synaptic plasticity and GTPase activity.

## Author Contributions

LI conducted research, analyzed data, wrote the paper, and constructed illustrations. AK performed experiments, contributed in writing the manuscript, and construction of figures. AR analyzed data. TF designed the experiments, analyzed data, and wrote the manuscript.

## Conflict of Interest Statement

The authors declare that the research was conducted in the absence of any commercial or financial relationships that could be construed as a potential conflict of interest.
