# Supplementary material for: DNA Methylation of Synaptic Genes in the Prefrontal Cortex Is Associated with Aging and Age-Related Cognitive Impairment
Source: Front Aging Neurosci. 2017 Aug 2;9:249. doi: 10.3389/fnagi.2017.00249 (PMC5539085; doi:10.3389/fnagi.2017.00249)
Supplement: Supplementary file 1 [file Table_1.PDF]

Supplementary Table 1. Age-related hypermethylation of genes within GO clusters in the mPFC

Gene Symbol	Gene Name	Postsynaptic density	GTPase activity
Dlgap1	DLG associated protein 1	X	
Clstn2	calsyntenin 2	X	
Erbb4	erb-b2 receptor tyrosine kinase 4	X	
Exoc4	exocyst complex component 4	X	
Grik2	glutamate ionotropic receptor kainate type subunit 2	X	
Grm5	glutamate metabotropic receptor 5	X	
Grm1	glutamate receptor, metabotropic 1	X	
Nlgn1	neuroligin 1	X	
Park2	parkin RBR E3 ubiquitin protein ligase	X	
Kcnd2	potassium voltage-gated channel subfamily D member 2	X	
Ppp1r9a	protein phosphatase 1, regulatory subunit 9A	X	
Agap2	ArfGAP with GTPase domain, ankyrin repeat and PH domain 2		X
Arhgef9	Cdc42 guanine nucleotide exchange factor 9		X
Dennd1b	DENN domain containing 1B		X
Epha3	Eph receptor A3		X
Farp1	FERM, ARH/RhoGEF and pleckstrin domain protein 1		X
Rabgap1l	RAB GTPase activating protein 1-like		X
Rasal2	RAS protein activator like 2		X
Ralgapa1	Ral GTPase activating protein catalytic alpha subunit 1		X
Arhgap10	Rho GTPase activating protein 10		X
Sbf2	SET binding factor 2		X
Srgap3	SLIT-ROBO Rho GTPase activating protein 3		X
Tbc1d15	TBC1 domain family, member 15		X
Tbc1d5	TBC1 domain family, member 5		X
Dock3	dedicator of cyto-kinesis 3		X
Dock9	dedicator of cytokinesis 9		X
Elmo1	engulfment and cell motility 1		X
Prkg1	protein kinase, cGMP-dependent, type 1		X
