# Supplementary material for: DNA Methylation of Synaptic Genes in the Prefrontal Cortex Is Associated with Aging and Age-Related Cognitive Impairment
Source: Front Aging Neurosci. 2017 Aug 2;9:249. doi: 10.3389/fnagi.2017.00249 (PMC5539085; doi:10.3389/fnagi.2017.00249)
Supplement: Supplementary file 2 [file Table_2.PDF]

Supplementary Table 2. Age-related differentially methylated CpGs correlated to RNA levels (Chrom – Chromosome; Pos – Position; Dec – Decreased; Inc – Increased)

Chrom & Pos	Gene Name	Genomic Feature	Direction of DNA	DNA RNA R value	Aged N	Aged depth	Young N	Young depth
**chr5-63114733**	*Unc13b*	Intron	Dec	-0.84	5	33	3	18
**chr1-65425484**	*Vom2r16*	Intron	Dec	-0.68	9	53	3	21
**chr5-163257661**	*Crocc*	Exon	Inc	-0.67	3	16	3	19
**chr3-14271337**	*Sh3glb2*	Intron	Dec	-0.67	8	53	6	37
**chrX-109119203**	*Il1rapl2*	Intron	Dec	-0.64	8	58	4	23
**chr13-51463293**	*Thsd7b*	Intron	Inc	-0.64	8	53	6	37
**chr1-26351829**	*LOC100361380*	Intron	Inc	-0.63	6	39	3	19
**chr17-90214440**	*Gpr158*	Intron	Dec	-0.61	4	21	3	20
**chr9-7456587**	*LOC100360856*	Intron	Inc	-0.59	6	49	3	26
**chr20-55666422**	*Grik2*	Intron	Inc	-0.58	8	68	3	21
**chr1-13505401**	*AABR06000596.1*	Intron	Dec	-0.56	12	96	3	25
**chr6-83492681**	*Akap6*	Intron	Inc	-0.56	11	75	3	20
**chr12-7650654**	*Fry*	Intron	Inc	-0.55	9	51	5	33

**chr13-61223431**	*Dennd1b*	Intron	Inc	-0.55	13	88	5	43
**chr10-96478278**	*Cep112*	Intron	Inc	-0.55	8	48	3	18
**chr8-108450234**	*Stag1*	Intron	Dec	-0.54	11	68	4	30
**chr13-51462477**	*Thsd7b*	Intron	Inc	-0.53	15	104	9	69
**chr19-70702453**	*Pard3*	Intron	Dec	-0.52	8	56	4	27
**chr4-227688126**	*Clec2dl1*	Intron	Inc	-0.50	16	140	7	58
**chr5-139550170**	*Zswim5*	Intron	Inc	-0.50	6	35	4	31
**chr12-1491162**	*5S_rRNA*	Exon	Dec	-0.50	8	59	3	23
**chr5-33238917**	*Necab1*	Intron	Inc	-0.49	10	79	7	54
**chr2-109001537**	*Ralyl*	Intron	Inc	-0.49	7	43	5	42
**chr4-137695732**	*Tpk1*	Intron	Dec	-0.49	12	137	4	56
**chr19-39901210**	*AABR06098126.1*	Intron	Inc	-0.49	5	29	4	32
**chr12-6464425**	*Vom2r60*	Intron	Dec	-0.48	7	42	5	34
**chr2-113371933**	*Chmp4c*	Intron	Dec	-0.48	9	63	4	21
**chr4-29957195**	*Ppp1r9a*	ExonIntron	Inc	-0.48	5	30	3	17

**chr1-6693136**	*Grm1*	Intron	Inc	-0.48	13	86	6	40
**chr2-148258660**	*Sclt1*	Intron	Inc	-0.47	10	70	4	25
**chr13-51462457**	*Thsd7b*	Intron	Inc	-0.47	14	103	8	69
**chr1-256639470**	*Prkg1*	Intron	Inc	-0.47	8	50	3	22
**chrX-15177103**	*Lancl3*	Intron	Inc	-0.47	5	34	4	26
**chr3-165917589**	*Jph2*	Intron	Dec	-0.47	5	38	3	19
**chr12-22404134**	*AABR06070908.1*	Intron	Dec	-0.46	5	29	3	16
**chr4-133773929**	*Mgam*	Intron	Inc	-0.46	16	126	5	33
**chr1-13505239**	*AABR06000596.1*	Intron	Dec	-0.46	9	72	4	31
**chr12-41683448**	*Tctn1*	Intron	Dec	-0.46	9	65	5	33
**chr6-109803622**	*Fut8*	Intron	Dec	-0.45	6	43	3	18
**chr2-18688285**	*Xrcc4*	Intron	Dec	-0.45	10	63	3	16
**chr8-126512442**	*Itga9*	Intron	Dec	-0.45	13	82	6	37
**chr17-48613491**	*Sugct*	Intron	Dec	-0.44	9	75	7	43
**chr9-61099227**	*Ankrd44*	Intron	Dec	-0.44	8	49	3	15

**chr6-104361262**	*Ccdc175*	Intron	Dec	-0.44	18	295	9	125
**chr4-233285565**	*RGD1306151*	Exon	Inc	-0.44	6	47	7	48
**chr19-463684**	*AABR06096743.1*	Exon	Dec	-0.43	10	79	4	38
**chr9-7420092**	*LOC10036085 6*	Intron	Dec	-0.43	11	94	8	51
**chr8-131099822**	*Zfp167*	Intron	Dec	-0.43	16	138	8	67
**chr16-2514289**	*Asb14*	Intron	Dec	-0.42	6	42	5	30
**chr16-57761299**	*Sgcz*	Intron	Dec	-0.42	14	101	7	50
**chr9-50199264**	*RGD1305645*	Intron	Dec	-0.41	10	84	4	26
**chr1-158069854**	*Grm5*	Intron	Inc	-0.41	5	39	3	21
**chr6-92639171**	*Lrfn5*	Intron	Inc	-0.40	5	28	4	20
**chr3-120127582**	*Frmd5*	Intron	Dec	-0.40	10	75	4	24
**chr20-23487226**	*RGD1306739*	ExonIntron	Dec	-0.40	19	140	7	53
**chr1-13506494**	*AABR06000596.1*	Intron	Dec	-0.40	11	103	3	33
**chr1-241608497**	*Trpm6*	Intron	Dec	-0.40	14	101	3	16
**chr8-93628221**	*Ube3d*	Intron	Dec	-0.39	7	55	3	17

**chr19-67048450**	*5S_rRNA*	Exon	Dec	-0.38	20	786	10	802
**chr19-67048512**	*5S_rRNA*	Promoter	Dec	-0.38	20	1053	10	979
**chr19-67048539**	*5S_rRNA*	Promoter	Dec	-0.38	20	847	10	742
**chr2-175410131**	*Ssr3*	Intron	Dec	-0.38	17	126	6	33
**chr19-67048432**	*5S_rRNA*	Exon	Dec	-0.37	20	979	10	905
**chr1-13506654**	*AABR06000596.1*	Intron	Dec	-0.37	13	108	5	38
**chr19-67048465**	*5S_rRNA*	Exon	Dec	-0.37	20	432	10	459
**chr4-154067896**	*Fam13a*	Intron	Dec	-0.37	11	70	5	31
**chr19-67048585**	*5S_rRNA*	Promoter	Dec	-0.37	14	151	10	192
**chr19-67048466**	*5S_rRNA*	Exon	Dec	-0.37	20	1160	10	1092
**chr19-342492**	*LOC100912892*	Intron	Inc	-0.36	15	200	6	72
**chr19-67048535**	*5S_rRNA*	Promoter	Dec	-0.36	20	862	10	760
**chr2-260544728**	*Ppp3ca*	Intron	Inc	0.36	11	77	4	26
**chr1-28955567**	*AABR06001371.1*	Intron	Dec	0.37	14	110	6	57
**chr19-39876764**	*AABR06098126.1*	Intron	Dec	0.37	13	155	5	38

**chr19-38818714**	*LOC100359783*	Intron	Dec	0.37	9	80	3	27
**chr9-77288803**	*Spag16*	Intron	Dec	0.37	10	57	4	23
**chr14-46702753**	*LOC257642*	Intron	Dec	0.38	20	237	10	169
**chr3-166709795**	*Stk4*	Intron	Dec	0.38	7	45	4	30
**chr5-64828520**	*Zcchc7*	Intron	Inc	0.38	9	75	3	24
**chr6-38695100**	*Ncoa1*	Intron	Inc	0.38	4	23	3	15
**chr10-4265259**	*Emp2*	ExonIntron	Inc	0.39	11	72	4	30
**chr14-46702328**	*LOC257642*	Intron	Dec	0.39	20	833	10	475
**chr16-17573194**	*Sh2d4b*	ExonIntron	Inc	0.39	6	43	4	26
**chr3-120905161**	*Slc28a2*	Exon	Dec	0.39	6	44	3	19
**chr14-36339461**	*Scfd2*	Intron	Dec	0.39	10	77	7	43
**chr1-192732090**	*Sox6*	Intron	Inc	0.39	7	52	5	35
**chr8-41139559**	*AABR06054289.1*	Exon	Dec	0.39	9	81	3	32
**chr14-46702776**	*LOC257642*	Intron	Dec	0.40	15	116	8	78
**chr14-46702762**	*LOC257642*	Intron	Dec	0.40	19	208	10	143

**chr14-46834543**	*AABR06078903.1*	Intron	Dec	0.41	14	202	6	87
**chr5-173266603**	*Nphp4*	Intron	Dec	0.42	7	44	3	19
**chrX-11871589**	*Med14*	Intron	Dec	0.43	9	66	7	48
**chr3-147506141**	*Kiz*	Intron	Dec	0.43	6	40	3	17
**chr10-18304602**	*Ranbp17*	Intron	Inc	0.43	9	50	3	15
**chr5-176291236**	*LOC100362748*	Intron	Inc	0.43	7	49	5	37
**chr15-9505112**	*LOC100364581*	Intron	Inc	0.43	10	93	3	28
**chr1-126954960**	*Fam189a1*	Intron	Inc	0.44	7	43	3	16
**chr10-46153386**	*Pemt*	Intron	Dec	0.44	5	35	3	18
**chr13-39644448**	*Clasp1*	Intron	Dec	0.45	11	73	4	25
**chr4-60547314**	*Exoc4*	Intron	Dec	0.45	7	47	3	17
**chr3-42871412**	*Neb*	Intron	Dec	0.45	6	34	3	15
**chr9-7647305**	*AABR06058265.1*	Exon	Dec	0.45	7	53	3	19
**chr14-46702701**	*LOC257642*	Intron	Dec	0.45	17	136	10	106
**chr9-38320159**	*Prim2*	Intron	Inc	0.45	7	39	4	31

**chr9-7412833**	*LOC100360856*	Intron	Inc	0.45	3	19	4	24
**chr9-11435737**	*AABR06058610.1*	Intron	Dec	0.46	16	170	5	49
**chr14-2532285**	*Ccdc18*	Intron	Inc	0.46	14	102	7	57
**chr14-46702193**	*LOC257642*	Intron	Dec	0.46	19	284	9	136
**chr1-182276873**	*Sbf2*	Intron	Inc	0.46	18	163	9	103
**chr7-58219846**	*Tbc1d15*	Intron	Dec	0.47	10	63	8	57
**chr7-20567186**	*LOC300308*	Intron	Inc	0.47	5	28	3	21
**chr4-189491314**	*Ccdc174*	Intron	Inc	0.47	7	50	4	33
**chr6-39261366**	*LOC257642*	Intron	Dec	0.48	5	35	5	49
**chr15-113593705**	*Itgbl1*	Intron	Dec	0.50	8	49	4	25
**chr14-46702196**	*LOC257642*	Intron	Dec	0.51	19	286	9	139
**chr14-46834644**	*AABR06078903.1*	Intron	Dec	0.52	9	78	6	54
**chr17-47451361**	*Pou6f2*	Intron	Dec	0.52	3	23	3	17
**chr14-46702785**	*LOC257642*	Intron	Dec	0.53	17	165	9	98
**chr14-46702792**	*LOC257642*	Intron	Dec	0.54	9	61	5	39

**chr3-60264722**	*Xirp2*	Intron	Inc	0.54	13	124	4	31
**chr13-27448648**	*Cntnap5b*	Intron	Inc	0.60	7	39	4	25
**chr7-12605871**	*Dos*	Exon	Dec	0.61	8	49	3	16
**chr2-2982624**	*Ttc37*	Intron	Dec	0.61	8	56	4	24
**chr9-7414403**	*LOC100360856*	Intron	Dec	0.62	8	65	5	36
**chr14-91851642**	*Grb10*	Intron	Inc	0.62	7	45	3	16
**chr1-26345148**	*LOC100361380*	Intron	Dec	0.63	13	168	4	41
**chr4-141196987**	*LOC100364190*	Intron	Dec	0.64	20	369	9	155
**chr4-45229758**	*Cav1*	Intron	Inc	0.69	4	25	4	23
**chr1-204961410**	*Eif3c*	Promoter	Inc	0.69	13	120	4	43
**chr1-75575066**	*Rdh13*	Intron	Dec	0.70	11	75	5	36
**chr18-37648862**	*Jakmip2*	Intron	Dec	0.71	8	57	4	21
**chr19-39208258**	*RGD1562877*	Intron	Inc	0.79	16	135	3	46
**chr6-85973536**	*Ralgapa1*	Intron	Inc	0.79	4	26	4	30
