# Supplementary material for: DNA Methylation of Synaptic Genes in the Prefrontal Cortex Is Associated with Aging and Age-Related Cognitive Impairment
Source: Front Aging Neurosci. 2017 Aug 2;9:249. doi: 10.3389/fnagi.2017.00249 (PMC5539085; doi:10.3389/fnagi.2017.00249)
Supplement: Supplementary file 3 [file Table_3.PDF]

Supplementary Table 3. Hypermethylation of genes within GO clusters of the mPFC during delayed shifting

Gene Symbol	Gene Name	Synapse	Postsynaptic density	Ion channel activity
Dnajc6	DnaJ heat shock protein family	X	X	
Erc2	ELKS/RAB6-interacting/CAST family member 2	X	X	
Fchsd2	FCH and double SH3 domains 2	X		
Gsg1l	GSG1-like	X		
Amph	amphiphysin	X		
Ank3	ankyrin 3	X		
Anks1b	ankyrin repeat and sterile alpha motif domain containing 1B	X	X	
Cacnb4	calcium voltage-gated channel auxiliary subunit beta 4	X		X
Clstn2	calsyntenin 2	X	X	
Ctnna2	catenin alpha 2	X	X	
Cep112	centrosomal protein 112	X		
Dpysl2	dihydropyrimidinase-like 2	X		
Dlg2	discs large MAGUK scaffold protein 2	X	X	
DMD	dystrophin	X		
Egfr	epidermal growth factor receptor	X		
Erbb4	erb-b2 receptor tyrosine kinase 4	X	X	
Esr1	estrogen receptor 1	X		
Exoc4	exocyst complex component 4	X	X	
Gabra3	gamma-aminobutyric acid type A receptor alpha3 subunit	X		X
Gria4	glutamate ionotropic receptor AMPA type subunit 4	X	X	X
Grin2b	glutamate ionotropic receptor NMDA type subunit 2B	X	X	X
Grik1	glutamate ionotropic receptor kainate type subunit 1	X	X	X

Grik2	glutamate ionotropic receptor kainate type subunit 2	X	X	X
Grip1	glutamate receptor interacting protein 1	X	X	
Insr	insulin receptor	X		
Lrfn1	leucine rich repeat and fibronectin type III domain containing 1	X	X	
Musk	muscle associated receptor tyrosine kinase	X		
Nlgn1	neuroligin 1	X	X	
Nos1	nitric oxide synthase 1	X	X	
Ophn1	oligophrenin 1	X		
Park2	parkin RBR E3 ubiquitin protein ligase	X	X	
Pclo	piccolo	X	X	
Ppp1r9a	protein phosphatase 1, regulatory subunit 9A	X	X	
Rims2	regulating synaptic membrane exocytosis 2	X		
Sorcs3	sortilin-related VPS10 domain containing receptor 3	X	X	
Synj1	synaptojanin 1	X		
Snap91	synaptosomal-associated protein 91	X	X	
Tenm2	teneurin transmembrane protein 2	X		
Vamp7	vesicle-associated membrane protein 7	X		
Zmynd8	zinc finger, MYND-type containing 8	X		
Ano5	anoctamin 5			X
Ano6	anoctamin 6			X
Cacnb2	calcium voltage-gated channel auxiliary subunit beta 2			X
Cacng3	calcium voltage-gated channel auxiliary subunit gamma 3			X
Cacna1e	calcium voltage-gated channel subunit alpha1 E			X
Cftr	cystic fibrosis transmembrane conductance regulator			X
Il1rapl1	interleukin 1 receptor accessory protein-like 1			X

Mcoln1	mucolipin 1	X
Piezo1	piezo-type mechanosensitive ion channel component 1	X
Kcnip3	potassium voltage-gated channel interacting protein 3	X
Kcnip4	potassium voltage-gated channel interacting protein 4	X
Kcnab1	potassium voltage-gated channel subfamily A member regulatory beta subunit 1	X
Kcnh8	potassium voltage-gated channel subfamily H member 8	X
Nalcn	sodium leak channel, non-selective	X
Slc24a3	solute carrier family 24 member 3	X
Trpc1	transient receptor potential cation channel, subfamily C, member 1	X
Trpc4	transient receptor potential cation channel, subfamily C, member 4	X
Trpc5	transient receptor potential cation channel, subfamily C, member 5	X
Tpcn1	two pore segment channel 1	X
