# Supplementary material for: DNA Methylation of Synaptic Genes in the Prefrontal Cortex Is Associated with Aging and Age-Related Cognitive Impairment
Source: Front Aging Neurosci. 2017 Aug 2;9:249. doi: 10.3389/fnagi.2017.00249 (PMC5539085; doi:10.3389/fnagi.2017.00249)
Supplement: Supplementary file 4 [file Table_4.PDF]

Supplementary Table 4. Hypomethylation of genes within GO clusters of the mPFC during delayed shifting

Gene Symbol	Gene Name	Neuron part	Cytoskeleton organization
Htr2a	5-hydroxytryptamine receptor 2A	X	
Brinp3	BMP/retinoic acid inducible neural specific 3	X	
Dcc	DCC netrin 1 receptor	X	
Dlgap1	DLG associated protein 1	X	
Polg	DNA polymerase gamma, catalytic subunit	X	
Dnajc6	DnaJ heat shock protein family	X	
Elmod3	ELMO domain containing 3	X	
Ephb1	Eph receptor B1	X	
Gpr149	G protein-coupled receptor 149	X	
Rab3c	RAB3C, member RAS oncogene family	X	
Ric3	RIC3 acetylcholine receptor chaperone	X	
Rapgef2	Rap guanine nucleotide exchange factor 2	X	
Tbc1d24	TBC1 domain family, member 24	X	
Acadm	acyl-CoA dehydrogenase, C-4 to C-12 straight chain	X	
Ahcyl2	adenosylhomocysteinase-like 2	X	
Adcy2	adenylate cyclase 2	X	
Agtr1a	angiotensin II receptor, type 1a	X	
Ank3	ankyrin 3	X	X
Anks1b	ankyrin repeat and sterile alpha motif domain containing 1B	X	
Atg5	autophagy related 5	X	
Begain	brain-enriched guanylate kinase-associated	X	
Cdh13	cadherin 13	X	
Cacna1d	calcium voltage-gated channel subunit alpha1 D	X	
Ctnna2	catenin alpha 2	X	
Cep290	centrosomal protein 290	X	
Cobl	cordon-bleu WH2 repeat protein	X	X
Cngb3	cyclic nucleotide gated channel beta 3	X	
Cyfip1	cytoplasmic FMR1 interacting protein 1	X	X
Dmd	dystrophin	X	X
Erbb4	erb-b2 receptor tyrosine kinase 4	X	
Esr1	estrogen receptor 1	X	
Exoc4	exocyst complex component 4	X	

Gabrb1	gamma-aminobutyric acid type A receptor beta 1 subunit	X	
Grin2b	glutamate ionotropic receptor NMDA type subunit 2B	X	
Grm8	glutamate metabotropic receptor 8	X	
Grxcr1	glutaredoxin and cysteine rich domain containing 1	X	
Glrb	glycine receptor, beta	X	
Ghr	growth hormone receptor	X	
Inha	inhibin alpha subunit	X	
Il1rapl1	interleukin 1 receptor accessory protein-like 1	X	
Katna1	katanin catalytic subunit A1	X	X
Kirrel	kin of IRRE like	X	X
Lama2	laminin subunit alpha 2	X	
Mapk10	mitogen activated protein kinase 10	X	
Mbp	myelin basic protein	X	
Myo3a	myosin IIIA	X	
Nfasc	neurofascin	X	
Nlgn1	neuroligin 1	X	X
Ncoa1	nuclear receptor coactivator 1	X	
Ophn1	oligophrenin 1	X	X
Pard3	par-3 family cell polarity regulator	X	X
Pde1a	phosphodiesterase 1A	X	
Pde1c	phosphodiesterase 1C	X	
Pde4b	phosphodiesterase 4B	X	
Plcb4	phospholipase C, beta 4	X	
Pclo	piccolo	X	X
Kcnip3	potassium voltage-gated channel interacting protein 3	X	
Kcnip4	potassium voltage-gated channel interacting protein 4	X	
Pcdh15	protocadherin 15	X	X
Reln	reelin	X	
Rims2	regulating synaptic membrane exocytosis 2	X	
Rgs7	regulator of G-protein signaling 7	X	
Scgn	secretagogin, EF-hand calcium binding protein	X	
Slc4a10	solute carrier family 4 member 10	X	
Spta1	spectrin, alpha, erythrocytic 1	X	X
Ston2	stonin 2	X	
Sv2b	synaptic vesicle glycoprotein 2b	X	
Syt17	synaptotagmin 17	X	

Wls	wntless Wnt ligand secretion mediator	X	
Zmynd8	zinc finger, MYND-type containing 8	X	
Cdc42bpa	CDC42 binding protein kinase alpha		X
Epha3	Eph receptor A3		X
Fer	FER tyrosine kinase		X
Frmd3	FERM domain containing 3		X
Frmd5	FERM domain containing 5		X
Fry	FRY microtubule binding protein		X
Rock1	Rho-associated coiled-coil containing protein kinase 1		X
Slain2	SLAIN motif family, member 2		X
Tnik	TRAF2 and NCK interacting kinase		X
Wdpcp	WD repeat containing planar cell polarity effector		X
Chmp3	charged multivesicular body protein 3		X
Cluap1	clusterin associated protein 1		X
Ccdc13	coiled-coil domain containing 13		X
Ccdc151	coiled-coil domain containing 151		X
Diaph2	diaphanous-related formin 2		X
Dnah5	dynein, axonemal, heavy chain 5		X
Dnah7	dynein, axonemal, heavy chain 7		X
Elmo1	engulfment and cell motility 1		X
Efna5	ephrin A5		X
Fign	fidgetin, microtubule severing factor		X
Kif4a	kinesin family member 4A		X
Pibf1	progesterone immunomodulatory binding factor 1		X
Prkce	protein kinase C, epsilon		X
Ptk2	protein tyrosine kinase 2		X
Ptpn1	protein tyrosine phosphatase, non-receptor type 1		X
Ptpn21	protein tyrosine phosphatase, non-receptor type 21		X
Sdccag8	serologically defined colon cancer antigen 8		X
LOC688970	similar to serine/threonine kinase		X
Spag16	sperm associated antigen 16		X
Synpo2	synaptopodin 2		X
Sdcbp	syndecan binding protein		X
Tnks	tankyrase		X
Ttc17	tetratricopeptide repeat domain 17		X
Trdn	triadin		X

Tuba1a	tubulin, alpha 1A	X
Ulk4	unc-51 like kinase 4	X
Vasp	vasodilator-stimulated phosphoprotein	X
Xirp2	xin actin-binding repeat containing 2	X
Zmym4	zinc finger MYM-type containing 4	X
