# Supplementary material for: DNA Methylation of Synaptic Genes in the Prefrontal Cortex Is Associated with Aging and Age-Related Cognitive Impairment
Source: Front Aging Neurosci. 2017 Aug 2;9:249. doi: 10.3389/fnagi.2017.00249 (PMC5539085; doi:10.3389/fnagi.2017.00249)
Supplement: Supplementary file 5 [file Table_5.PDF]

Supplementary Table 5. CpGs correlation to set shifting and RNA levels (Chrom – Chromosome; Pos – Position)

Chrom & Pos	Gene Name	Genomic Feature	DNA Set Shift R value	DNA RNA R value	AI N	AI depth	AU N	AU depth
**chr14-12698040**	*LOC685158*	Intron	-0.62	-0.98	3	22	3	19
**chr1-238604286**	*Tle4*	Intron	0.47	-0.95	3	18	3	20
**chr13-27285285**	*Cntnap5b*	Intron	-0.68	-0.90	3	19	3	18
**chr13-62056259**	*Cfh*	Intron	0.52	-0.89	3	21	4	32
**chr10-36072106**	*Adamts2*	Exon	-0.56	-0.88	3	21	3	16
**chr9-53368402**	*Mfsd6*	Intron	0.50	-0.87	3	22	3	17
**chr19-416459**	*LOC100912892*	Intron	-0.54	-0.86	3	18	3	19
**chr11-72222723**	*Mylk*	Intron	-0.63	-0.85	3	17	4	27
**chr2-143805843**	*Spata5*	Intron	-0.58	-0.85	3	22	4	34
**chr6-134713974**	*Tc2n*	Intron	-0.82	-0.84	3	19	3	25
**chr2-134027247**	*Tnik*	Exon	-0.62	-0.83	3	17	3	17
**chrX-69086319**	*Ophn1*	Intron	0.61	-0.83	3	17	4	23
**chr1-225377700**	*Ighmbp2*	Intron	0.45	-0.83	3	20	3	19
**chr2-205109270**	*Lrba*	Intron	-0.53	-0.83	3	19	3	20

**chrX-109119203**	*Il1rapl2*	Intron	0.86	-0.83	5	27	3	31
**chr5-134666161**	*AABR06039340.1*	Intron	-0.47	-0.82	3	24	3	18
**chr14-106613535**	*Wdpcp*	Intron	-0.57	-0.81	3	45	4	35
**chr20-32195549**	*Unc5b*	Intron	0.66	-0.81	3	19	3	15
**chr1-272811877**	*Fbxw4*	Intron	-0.52	-0.80	3	17	3	18
**chr8-80606729**	*Wdr72*	Exon	0.55	-0.79	3	20	4	24
**chr19-39003252**	*AABR06097983.1*	Intron	0.80	-0.79	3	20	3	24
**chr1-204962126**	*Eif3c*	Promoter	0.56	-0.79	3	20	3	26
**chr1-82642117**	*Zfp428*	Exon	0.80	-0.79	3	23	3	20
**chr2-191651490**	*Schip1*	Intron	-0.64	-0.78	4	30	4	25
**chr9-7455347**	*LOC100360856*	Intron	0.50	-0.77	5	43	7	59
**chr19-70702501**	*Pard3*	Intron	-0.64	-0.77	5	37	3	15
**chr4-184301437**	*Vom1r90*	Intron	0.77	-0.77	3	17	3	17
**chr7-78011941**	*Rims2*	Intron	-0.71	-0.76	6	35	5	34
**chr1-6193611**	*Adgb*	Intron	0.72	-0.76	3	20	4	35
**chr18-37540297**	*Jakmip2*	Intron	0.56	-0.75	4	20	6	40

**chr1-85221649**	*BC024978*	Intron	-0.80	-0.75	3	20	4	23
**chr7-569405**	*AABR06046926.1*	Promoter	-0.83	-0.75	3	27	5	35
**chr9-35899643**	*Phf3*	Intron	0.76	-0.75	5	29	6	36
**chr9-11439292**	*AABR06058610.1*	Intron	0.66	-0.74	3	21	5	34
**chr6-51670725**	*Greb1*	Exon	0.57	-0.74	3	15	3	16
**chr2-222894626**	*Ptgfrn*	Intron	0.63	-0.74	4	22	3	18
**chr6-112109242**	*LOC680519*	Promoter	-0.62	-0.74	3	32	6	43
**chr2-3074559**	*Fam81b*	Intron	0.74	-0.73	4	22	6	37
**chr1-13506637**	*AABR06000596.1*	Intron	0.49	-0.73	6	43	6	56
**chr5-2349760**	*Stau2*	Intron	0.64	-0.73	4	22	3	16
**chr4-42377148**	*Cftr*	Intron	0.67	-0.73	5	28	3	19
**chr10-44989356**	*Zfp39*	Intron	-0.80	-0.73	4	27	3	21
**chr1-182277037**	*Sbf2*	Intron	0.65	-0.73	4	22	4	21
**chr6-67156639**	*Dgkb*	Intron	-0.45	-0.72	3	18	3	19
**chr9-1364876**	*Tbc1d5*	Intron	0.82	-0.72	4	29	4	23
**chr9-77464701**	*Spag16*	Intron	-0.57	-0.72	4	21	4	26

**chr18-78537598**	*Zfp236*	Intron	0.51	-0.72	3	18	3	18
**chr11-61533588**	*Zbtb20*	Intron	0.68	-0.72	4	22	4	26
**chr3-60264614**	*Xirp2*	Intron	0.68	-0.72	6	43	8	70
**chr1-42776221**	*Esr1*	Intron	-0.72	-0.72	5	50	8	59
**chr4-189491324**	*Ccdc174*	Intron	0.81	-0.71	3	23	3	27
**chr9-61351094**	*Coq10b*	Intron	-0.48	-0.71	4	25	4	32
**chr6-9572613**	*Prkce*	Intron	-0.95	-0.70	3	17	3	18
**chr1-26397237**	*LOC100361380*	Intron	-0.54	-0.70	3	21	6	51
**chr1-221531475**	*Muc6*	ExonIntron	-0.62	-0.70	4	35	3	21
**chr1-177221868**	*RGD1561034*	Exon	0.85	-0.70	3	17	3	20
**chr10-105470140**	*Mfsd11*	Intron	-0.50	-0.70	5	32	3	17
**chr5-148654743**	*Zmym4*	Exon	0.52	-0.69	4	31	3	22
**chr14-66769485**	*Kcnip4*	Intron	0.47	-0.69	4	22	6	43
**chr15-21660645**	*4930452B06Rik*	Intron	0.68	-0.69	4	22	4	26
**chr14-92199200**	*Cobl*	Intron	-0.78	-0.69	4	26	3	18

**chr9-37079745**	*Kcnh8*	Intron	0.55	-0.69	3	18	4	22
**chr4-38731402**	*Thsd7a*	Intron	0.81	-0.68	3	21	3	20
**chr1-159237599**	*Tmem135*	Intron	0.72	-0.68	5	35	5	32
**chr5-83218442**	*Col27a1*	Intron	-0.57	-0.68	4	32	3	19
**chr15-9694055**	*LOC680653*	Intron	0.51	-0.68	3	18	4	24
**chr8-41138225**	*AABR06054289.1*	Exon	-0.49	-0.68	3	23	4	25
**chr19-57695168**	*Wwox*	Intron	0.53	-0.67	3	19	4	20
**chr20-6853736**	*RT1-CE2*	Intron	-0.60	-0.67	3	24	3	24
**chr20-47223904**	*Cdk19*	Intron	0.45	-0.67	4	30	6	34
**chr15-58230065**	*Fndc3a*	Intron	0.53	-0.67	3	21	3	19
**chr4-227688157**	*Clec2dl1*	Intron	-0.55	-0.66	9	68	7	68
**chr15-111354048**	*Dock9*	Intron	0.59	-0.66	4	28	5	33
**chr8-23057843**	*Ccdc151*	Exon	-0.81	-0.66	3	19	3	16
**chr19-40076095**	*AABR06098126.1*	Intron	0.78	-0.66	5	43	3	28
**chr14-2532505**	*Ccdc18*	ExonIntron	-0.56	-0.66	6	43	6	55
**chr8-91134224**	*Bckdhb*	Intron	0.49	-0.66	6	36	4	28

**chr7-9946636**	*ENSRNOG00000051152*	Intron	0.92	-0.65	4	29	4	40
**chr9-50203898**	*RGD1305645*	Intron	-0.94	-0.65	3	16	3	18
**chr4-193856673**	*Fam19a4*	Intron	-0.53	-0.65	6	42	6	44
**chr2-143772832**	*Spata5*	Intron	-0.62	-0.65	4	26	4	32
**chr19-400438**	*LOC100912892*	Intron	-0.50	-0.64	4	30	5	44
**chr1-65331862**	*Vom2r16*	Intron	0.50	-0.64	4	29	7	46
**chr9-11435578**	*AABR06058610.1*	Intron	0.64	-0.64	4	41	5	42
**chr11-90446012**	*Lztr1*	Exon	-0.72	-0.64	3	28	4	29
**chr5-175752102**	*Mmel1*	Intron	0.72	-0.64	3	17	3	17
**chr9-111286875**	*Fer*	Intron	-0.46	-0.63	3	22	3	16
**chr9-113456209**	*Wash*	Intron	0.51	-0.63	3	21	3	17
**chr15-9558539**	*LOC100364581*	Intron	0.47	-0.62	3	25	3	19
**chr17-91120997**	*LOC688970*	Intron	0.50	-0.62	3	19	6	45
**chr20-5294288**	*RT1-T24-4*	Exon	-0.59	-0.62	5	36	5	41
**chr9-11299031**	*AABR06058583.1*	Exon	0.48	-0.62	3	18	4	38
**chr4-204617272**	*Sumf1*	Intron	-0.56	-0.62	5	31	3	26

**chr5-174858842**	*Tp73*	Intron	-0.64	-0.62	3	18	4	21
**chr7-23169547**	*LOC100361411*	Intron	-0.65	-0.62	3	25	4	33
**chr6-39261354**	*LOC257642*	Intron	0.45	-0.62	3	29	3	16
**chr1-204962080**	*Eif3c*	Promoter	0.96	-0.62	3	21	3	28
**chr14-62189838**	*Kctd8*	Intron	-0.57	-0.61	3	17	5	30
**chr10-96478246**	*Cep112*	Intron	0.51	-0.61	4	21	4	29
**chr5-62469277**	*Fam219a*	Intron	-0.88	-0.61	3	25	3	23
**chr2-143772872**	*Spata5*	Intron	-0.61	-0.61	4	37	4	41
**chr9-7457196**	*LOC100360856*	Intron	0.80	-0.61	3	23	3	16
**chr5-64362501**	*Melk*	Intron	-0.57	-0.61	3	22	3	17
**chr12-22079224**	*RGD1559588*	Intron	-0.48	-0.60	8	88	6	56
**chr19-322776**	*AABR06096719.1*	Intron	0.52	-0.60	3	16	3	19
**chr8-115073916**	*Dock3*	Intron	-0.85	-0.60	3	25	3	19
**chr4-193856428**	*Fam19a4*	Intron	-0.49	-0.60	5	34	6	43
**chr2-72261455**	*Ghr*	Intron	0.58	-0.59	5	38	7	47
**chr7-23168943**	*LOC100361411*	Exon	-0.63	-0.59	4	49	8	76

**chr5-60994046**	*Aco1*	Intron	0.52	-0.59	3	19	3	20
**chr6-120691171**	*Sptlc2*	Intron	0.58	-0.59	5	31	4	24
**chr4-151266095**	*Pde1c*	Intron	0.51	-0.58	4	26	5	37
**chr16-64294110**	*Fut10*	Intron	-0.62	-0.58	3	16	3	17
**chr2-114139394**	*Zfp704*	Intron	-0.62	-0.58	4	23	4	32
**chr18-66017457**	*Dcc*	Intron	-0.46	-0.58	3	17	4	26
**chr6-39260727**	*LOC257642*	Intron	0.49	-0.58	6	62	9	90
**chr7-20629427**	*LOC300308*	Intron	-0.56	-0.58	3	30	5	33
**chr3-160334236**	*Fam83d*	Intron	-0.54	-0.57	3	27	3	28
**chr1-172434656**	*Fchsd2*	Intron	0.74	-0.57	5	41	5	36
**chr17-92354592**	*Gpr137b*	Intron	-0.84	-0.57	3	17	3	17
**chr14-89518512**	*Abca13*	Intron	-0.50	-0.57	4	39	5	36
**chr14-12698019**	*LOC685158*	Intron	-0.82	-0.57	3	24	4	24
**chr9-1367778**	*Tbc1d5*	Intron	0.59	-0.57	8	149	9	167
**chr4-60559066**	*Exoc4*	Intron	0.45	-0.57	3	23	6	52

**chr9-51104228**	*Gulp1*	Intron	0.81	-0.57	3	27	3	24
**chr9-11425000**	*AABR06058610.1*	Intron	0.67	-0.57	4	35	4	25
**chr19-300594**	*AABR06096719.1*	ExonIntron	0.55	-0.56	3	35	5	51
**chr19-61656117**	*Cdh13*	Intron	0.49	-0.56	3	19	3	17
**chr15-9908549**	*Cd99l2*	Intron	-0.54	-0.56	6	65	7	62
**chr12-53240421**	*Ttc28*	Intron	-0.64	-0.56	4	27	3	17
**chr14-82551167**	*Poln*	Intron	-0.56	-0.56	3	16	4	22
**chr10-96478345**	*Cep112*	Intron	-0.50	-0.56	4	24	4	28
**chr1-28913233**	*AABR06001371.1*	Intron	0.68	-0.56	4	25	3	16
**chr2-164676974**	*Nbea*	Intron	0.65	-0.56	4	23	3	19
**chr1-192727382**	*Sox6*	Intron	0.45	-0.56	3	19	4	26
**chr9-11436097**	*AABR06058610.1*	Intron	0.50	-0.56	5	33	3	20
**chr9-7441491**	*LOC100360856*	Intron	0.59	-0.56	3	21	6	63
**chr8-66075932**	*Tle3*	Exon	-0.46	-0.56	5	38	3	19
**chr2-3074596**	*Fam81b*	Intron	0.46	-0.55	3	15	6	34
**chr19-40056671**	*AABR06098126.1*	Intron	0.52	-0.55	4	34	8	49
**chr3-9136369**	*Gpsm1*	Exon	-0.85	-0.55	4	28	3	18

**chr14-46836573**	*AABR06078903.1*	Intron	0.61	-0.55	7	66	6	43
**chr2-228216029**	*Chia*	Intron	-0.64	-0.55	3	17	3	16
**chr1-172040987**	*Fam168a*	ExonIntron	0.91	-0.55	3	20	3	19
**chr19-37384608**	*LOC100363633*	Intron	0.46	-0.55	8	123	8	129
**chr1-204962138**	*Eif3c*	Promoter	0.57	-0.55	3	17	3	25
**chr2-191619277**	*Schip1*	Intron	-0.50	-0.55	4	25	5	38
**chr7-23169500**	*LOC100361411*	Intron	-0.67	-0.54	3	27	4	34
**chr11-34735104**	*Synj1*	Intron	0.75	-0.54	3	19	3	17
**chr14-25465028**	*Epha5*	Intron	0.61	-0.54	7	50	8	68
**chr5-62122538**	*Ubap2*	Intron	-0.49	-0.53	5	38	3	19
**chr19-308416**	*AABR06096719.1*	Intron	0.55	-0.53	3	20	3	19
**chr13-100462039**	*AABR06076326.1*	Intron	-0.70	-0.53	4	23	5	33
**chr1-191002687**	*Pde3b*	Intron	0.48	-0.53	3	22	3	19
**chr16-35498216**	*Galntl6*	Intron	-0.45	-0.53	4	24	5	38
**chr1-149529967**	*Aff2*	Intron	0.47	-0.53	3	26	5	35

**chr19-26041120**	*AABR06097301.1*	Intron	-0.52	-0.52	4	27	4	22
**chr9-75324411**	*Erbb4*	Intron	-0.86	-0.52	3	19	3	19
**chr5-2349773**	*Stau2*	Intron	0.67	-0.52	4	20	3	15
**chr19-303837**	*AABR06096719.1*	Intron	0.47	-0.52	7	68	6	56
**chr2-8910250**	*Gpr98*	Intron	0.52	-0.52	6	48	7	44
**chr20-23487161**	*RGD1306739*	ExonIntron	0.73	-0.52	3	18	4	29
**chr17-37253537**	*Agtr1a*	Intron	-0.65	-0.52	3	20	3	18
**chr2-113371933**	*Chmp4c*	Intron	0.46	-0.52	4	25	5	38
**chr7-117789948**	*Arhgap39*	Exon	0.68	-0.52	3	25	3	17
**chr19-66029743**	*Piezo1*	Exon	0.82	-0.52	3	18	3	18
**chr20-23487345**	*RGD1306739*	ExonIntron	-0.59	-0.52	4	26	5	31
**chr1-26693596**	*Clvs2*	Intron	0.59	-0.52	9	80	7	51
**chr19-343643**	*LOC100912892*	ExonIntron	0.54	-0.52	5	41	6	57
**chr10-105470099**	*Mfsd11*	Intron	-0.84	-0.51	5	30	3	16
**chr1-148288574**	*Gabra3*	Intron	-0.94	-0.51	4	30	3	17
**chr20-6743568**	*RT1-CE7*	Intron	-0.76	-0.51	3	19	4	40
**chr15-9491943**	*LOC100364581*	Intron	0.64	-0.51	3	25	4	31

**chr15-113313498**	*Nalcn*	Intron	-0.65	-0.51	3	18	3	17
**chr7-126019176**	*Atxn10*	Intron	0.88	-0.51	3	19	3	22
**chrX-114187746**	*Trpc5*	Intron	0.46	-0.51	5	32	4	26
**chr2-164514876**	*Nbea*	ExonIntron	-0.77	-0.51	3	24	3	22
**chr3-164064269**	*Ptprt*	Intron	-0.45	-0.51	4	25	3	18
**chr20-6728308**	*RT1-CE7*	Intron	-0.46	-0.50	5	33	6	38
**chr5-81257420**	*Snx30*	Intron	0.75	-0.50	4	23	3	17
**chr6-112106815**	*LOC680519*	Exon	0.56	-0.50	3	26	4	23
**chr9-7483787**	*LOC100360856*	Intron	0.51	-0.50	3	17	5	29
**chr6-142110120**	*Begain*	Exon	-0.53	-0.50	3	18	3	18
**chr15-9490216**	*LOC100364581*	Intron	0.52	-0.50	4	29	4	34
**chr15-9865540**	*Cd99l2*	Intron	-0.75	-0.50	5	46	6	56
**chr14-106807252**	*Wdpcp*	Intron	-0.68	-0.50	3	21	3	16
**chr1-33696028**	*Tert*	Exon	-0.69	-0.50	4	30	3	20
**chr19-37394441**	*LOC100363633*	Intron	-0.50	-0.50	3	17	3	36
**chr5-151211650**	*Sync*	Intron	-0.60	-0.49	3	16	4	21

**chr20-22590203**	*Ank3*	Intron	0.56	-0.49	4	25	6	40
**chr20-22374967**	*Ank3*	Intron	-0.45	-0.49	3	22	5	31
**chr9-109632650**	*Efna5*	Intron	-0.48	-0.49	3	21	4	24
**chr15-9911325**	*Cd99l2*	Intron	0.67	-0.49	3	19	3	28
**chr1-115530929**	*Herc2*	Intron	0.61	-0.49	4	22	6	35
**chr1-13555978**	*LOC100909555*	Intron	0.55	-0.49	4	32	8	45
**chr1-26362921**	*LOC100361380*	Intron	0.49	-0.49	9	103	9	94
**chr9-7410305**	*LOC100360856*	Intron	0.74	-0.49	3	19	3	18
**chr2-92137358**	*Cdh12*	Intron	0.92	-0.49	3	16	3	19
**chr4-137674392**	*Tpk1*	Intron	0.54	-0.49	5	45	6	64
**chr9-7468697**	*LOC100360856*	Intron	0.58	-0.49	6	55	6	55
**chr4-27526657**	*RGD1306626*	Intron	-0.48	-0.49	4	24	5	32
**chr19-37275194**	*LOC296778*	Exon	0.57	-0.49	3	18	5	46
**chr15-43696151**	*Cdadc1*	Intron	0.69	-0.49	4	29	5	31
**chr11-74005944**	*Lmln*	Intron	-0.74	-0.48	6	32	7	45
**chr4-42439697**	*Cftr*	Intron	0.53	-0.48	3	26	5	36

**chr10-96759181**	*Cep112*	Intron	-0.89	-0.48	3	23	4	33
**chrX-3195101**	*Ormdl1*	Exon	-0.78	-0.48	5	40	3	17
**chr9-10679131**	*LOC688241*	Intron	0.50	-0.48	3	17	5	33
**chr19-314425**	*AABR06096719.1*	Intron	-0.70	-0.48	5	46	4	24
**chr15-91253619**	*Scel*	Intron	-0.79	-0.48	5	33	8	58
**chr19-37452841**	*LOC100363633*	Intron	-0.75	-0.48	4	39	4	26
**chr17-91122611**	*LOC688970*	Exon	0.55	-0.47	5	65	7	77
**chr1-26377811**	*LOC100361380*	Intron	0.49	-0.47	4	27	5	34
**chr4-234054440**	*Grin2b*	Intron	0.77	-0.47	3	19	3	22
**chr19-38816659**	*LOC100359783*	Exon	0.55	-0.47	3	26	6	64
**chr9-29404405**	*Col19a1*	Intron	0.49	-0.47	3	18	4	25
**chr9-29549956**	*Col19a1*	Intron	-0.54	-0.47	4	23	3	15
**chrX-56449113**	*Il1rapl1*	Intron	0.60	-0.47	3	19	3	21
**chr10-96478351**	*Cep112*	Intron	0.55	-0.47	4	25	4	25
**chr2-130811318**	*Nlgn1*	Intron	0.45	-0.47	5	34	4	25
**chr17-48612554**	*Sugct*	Intron	-0.65	-0.46	3	17	3	18

**chr15-113815530**	*Fgf14*	Intron	0.47	-0.46	3	18	5	45
**chr9-11353671**	*Rn50_9_0114.1*	Promoter	0.48	-0.46	3	22	4	26
**chr1-51497089**	*Igf2r*	Intron	0.51	-0.46	4	29	4	22
**chrX-63234373**	*Pola1*	Intron	0.49	-0.46	4	26	4	26
**chr1-26415949**	*LOC100361380*	Intron	-0.74	-0.46	3	21	4	26
**chr3-51927740**	*Rbms1*	Intron	-0.91	-0.46	3	16	3	15
**chr15-9488922**	*LOC100364581*	Intron	-0.55	-0.46	5	55	6	55
**chr4-178248923**	*Hk2*	Exon	0.66	-0.46	4	33	4	25
**chr1-101928303**	*Ptov1*	Exon	0.50	-0.46	4	30	4	28
**chr2-108908853**	*Ralyl*	Intron	0.49	-0.46	5	75	5	57
**chr15-9570365**	*LOC100364581*	ExonIntron	-0.51	-0.46	8	102	9	148
**chr17-2208839**	*Cntnap3*	Intron	0.63	-0.46	8	54	4	31
**chr3-171859442**	*Zfp64*	Intron	-0.73	-0.46	4	34	4	31
**chr13-97706396**	*Rgs7*	Intron	-0.75	-0.45	3	20	5	40
**chr18-30522175**	*AABR06094669.1*	Exon	-0.59	-0.45	4	27	4	26
**chr9-1394216**	*Tbc1d5*	Intron	0.65	-0.45	3	32	6	45

**chr13-12343070**	*Cntnap5c*	Intron	0.45	-0.45	5	37	7	49
**chr7-23170936**	*LOC100361411*	Intron	-0.53	-0.45	3	16	5	35
**chr17-51311506**	*Hecw1*	Intron	-0.45	-0.45	9	114	11	139
**chr15-9622580**	*LOC680653*	ExonIntron	0.83	-0.45	3	18	3	21
**chr19-54377552**	*Fa2h*	Intron	0.69	-0.45	3	23	3	25
**chr19-37397144**	*LOC100363633*	Intron	0.64	-0.45	3	26	4	28
**chr12-22507965**	*AABR06070908.1*	Intron	-0.65	-0.45	4	23	4	27
**chr1-213183097**	*Adam12*	Intron	0.51	0.44	7	56	8	90
**chr7-83419246**	*Pkhd1l1*	Intron	-0.46	0.45	3	19	4	22
**chr19-374881**	*LOC100912892*	Intron	-0.49	0.45	7	105	9	121
**chr19-68301797**	*Trim67*	Exon	0.67	0.45	3	23	3	18
**chr1-249447689**	*Pip5k1b*	Intron	-0.55	0.45	3	18	3	15
**chr2-240460188**	*Dpyd*	Intron	-0.50	0.45	3	24	5	31
**chr3-165838500**	*Tox2*	Exon	0.67	0.45	3	18	3	17
**chr15-9018030**	*Nid2*	Intron	-0.69	0.45	3	19	3	19
**chr19-39863500**	*AABR06098126.1*	Intron	-0.57	0.45	5	47	7	55

**chr9-7412846**	*LOC100360856*	Intron	-0.65	0.45	5	42	5	32
**chr19-43922075**	*Slc10a7*	Intron	0.51	0.45	3	19	3	21
**chr18-1371889**	*Rock1*	Intron	-0.45	0.45	4	37	3	29
**chr9-7409793**	*LOC100360856*	Intron	-0.79	0.45	3	20	5	38
**chr2-246634374**	*Synpo2*	Intron	-0.65	0.45	4	32	7	79
**chr3-98879436**	*Trim44*	Intron	-0.57	0.45	6	41	5	36
**chr16-25083259**	*March1*	Intron	0.59	0.45	8	66	8	78
**chr15-58230324**	*Fndc3a*	Intron	-0.54	0.45	5	33	6	36
**chr1-246285726**	*Gda*	Intron	0.52	0.45	4	28	4	26
**chr9-60631179**	*Ccdc150*	Intron	0.62	0.46	7	48	7	45
**chr4-173123195**	*Ctnna2*	Intron	-0.81	0.46	3	16	3	19
**chr14-46260556**	*LOC364157*	Intron	-0.62	0.46	7	58	7	57
**chr14-14534651**	*Fras1*	Intron	0.80	0.46	3	21	3	19
**chr19-26041154**	*AABR06097301.1*	Intron	0.46	0.46	4	27	5	27
**chr9-7413323**	*LOC100360856*	Intron	-0.54	0.46	4	35	6	51
**chr14-113925030**	*Eml6*	Intron	-0.50	0.46	5	33	5	27
**chr15-9595063**	*LOC680653*	Intron	0.54	0.47	5	31	6	45

**chr19-37464399**	*LOC100363633*	Intron	0.78	0.47	4	27	6	55
**chr2-191651311**	*Schip1*	Intron	0.59	0.47	4	31	4	23
**chr13-103277272**	*Cdc42bpa*	Intron	-0.78	0.47	5	42	5	42
**chr4-137696628**	*Tpk1*	Intron	-0.62	0.47	4	25	5	31
**chr3-102530406**	*AABR06025251.1*	Intron	-0.74	0.47	3	16	3	20
**chr2-205211651**	*Lrba*	Intron	-0.53	0.47	3	22	3	15
**chr2-104229510**	*March3*	Intron	0.78	0.47	3	15	3	19
**chr19-38795109**	*LOC100909409*	Exon	-0.70	0.47	6	90	9	98
**chr7-23168998**	*LOC100361411*	Exon	0.47	0.47	3	36	5	53
**chr1-13556131**	*LOC100909555*	Intron	0.52	0.47	5	57	5	41
**chr6-101036460**	*Klhdc1*	Intron	-0.57	0.47	5	32	5	29
**chr17-85222369**	*Plxdc2*	Intron	-0.46	0.47	3	19	5	32
**chr6-36071331**	*Supt7l*	Exon	0.63	0.48	4	24	3	16
**chr17-91075231**	*LOC688970*	Intron	-0.61	0.48	3	31	7	60
**chr19-39208357**	*RGD1562877*	Intron	-0.54	0.48	9	83	10	103

**chr3-60264722**	*Xirp2*	Intron	-0.69	0.48	4	34	9	90
**chr4-16634476**	*Pclo*	Intron	0.47	0.48	4	32	6	47
**chr10-14405641**	*Unkl*	ExonIntron	-0.70	0.48	5	35	4	21
**chr13-73074635**	*Hmcn1*	Intron	-0.50	0.48	9	129	11	114
**chr3-43116743**	*Cacnb4*	Intron	-0.55	0.49	4	29	6	44
**chrX-53314599**	*AABR06104465.1*	Intron	0.64	0.49	4	37	4	29
**chr2-72205202**	*Ghr*	Intron	-0.77	0.49	6	43	6	39
**chr15-9520337**	*LOC100364581*	Intron	0.45	0.49	8	100	8	100
**chr13-60201086**	*Ptprc*	Intron	0.73	0.49	3	19	4	24
**chr2-131072360**	*Nlgn1*	Intron	-0.86	0.49	4	34	3	22
**chr2-205865955**	*Kirrel*	Intron	-0.46	0.49	3	17	4	22
**chr6-67859868**	*Dgkb*	Intron	0.85	0.50	3	16	3	17
**chr10-10616731**	*Cluap1*	Intron	-0.45	0.50	9	536	11	581
**chr19-40231714**	*LOC100912892*	Exon	-0.58	0.50	3	36	8	76
**chr18-6119975**	*Psma8*	Intron	0.86	0.50	3	17	3	22
**chr3-43116097**	*Cacnb4*	Intron	-0.54	0.50	4	30	5	35

**chr15-9512206**	*LOC100364581*	Intron	-0.53	0.50	5	30	3	20
**chr8-102307459**	*Slc9a9*	Intron	0.54	0.50	7	48	9	78
**chr19-37508930**	*LOC100363633*	Intron	-0.53	0.50	8	107	8	129
**chr2-3714556**	*Mctp1*	Intron	0.53	0.50	4	39	7	56
**chr1-238604289**	*Tle4*	Intron	0.53	0.50	3	19	3	18
**chr16-54513208**	*Mtmr7*	Intron	-0.57	0.50	5	37	7	50
**chr1-81219682**	*Rsph6a*	Exon	-0.56	0.51	3	17	3	15
**chr13-47728835**	*Nckap5*	Intron	0.46	0.51	6	50	6	47
**chr1-271007236**	*Abcc2*	Intron	0.57	0.51	3	22	3	16
**chr5-133489115**	*Faf1*	Intron	0.84	0.51	3	19	4	22
**chr19-38798042**	*LOC100909409*	Exon	-0.82	0.51	3	24	4	37
**chr2-256399039**	*Tbck*	Intron	0.58	0.51	5	30	6	42
**chr2-65080670**	*Arl15*	Intron	-0.79	0.51	3	20	3	16
**chr7-99074755**	*Fer1l6*	Intron	-0.80	0.51	3	25	5	28
**chr9-25974711**	*LOC100361830*	Exon	0.50	0.51	3	16	3	17
**chr3-26748580**	*LOC502622*	Intron	0.59	0.51	3	30	8	55

**chr6-111983770**	*AABR06045117.2*	Exon	0.49	0.51	3	25	3	24
**chr1-26371514**	*LOC100361380*	Intron	-0.79	0.52	3	24	3	22
**chr19-37277869**	*LOC296778*	Exon	0.52	0.52	3	23	3	19
**chr12-3865856**	*Insr*	Intron	0.50	0.52	8	63	9	78
**chr1-26359639**	*LOC100361380*	Intron	0.50	0.52	3	20	5	30
**chr4-180432762**	*Dysf*	Intron	0.52	0.52	4	26	6	33
**chr5-33239170**	*Necab1*	Intron	-0.53	0.52	4	25	5	37
**chr18-30522131**	*AABR06094669.1*	Exon	0.59	0.52	5	29	4	26
**chr13-27008261**	*Cntnap5b*	Intron	-0.71	0.52	4	29	3	19
**chr9-68365175**	*Pard3b*	Intron	-0.71	0.52	3	17	4	28
**chr16-29790925**	*Ddx60*	Intron	0.82	0.53	3	19	3	17
**chr1-75711570**	*Ptprh*	Intron	0.82	0.53	3	16	3	19
**chr5-33359119**	*Necab1*	Intron	-0.76	0.53	4	36	5	43
**chr14-46196015**	*LOC364157*	Intron	0.45	0.53	5	30	3	20
**chr10-96478349**	*Cep112*	Intron	0.73	0.54	4	25	4	29

**chr6-104485557**	*Rtn1*	Intron	0.71	0.54	3	15	4	24
**chr4-164245325**	*Chmp3*	Intron	-0.58	0.54	4	21	5	27
**chr1-192727466**	*Sox6*	Intron	-0.51	0.54	4	30	7	36
**chr5-66090466**	*Tdrd7*	Intron	-0.57	0.54	3	33	3	20
**chr2-60694620**	*Rab3c*	Intron	-0.63	0.54	3	20	4	25
**chr13-73019089**	*Hmcn1*	Intron	-0.46	0.54	4	22	4	32
**chr5-119108282**	*Cyp2j10*	Intron	0.48	0.55	3	15	4	23
**chr1-149529807**	*Aff2*	Intron	-0.68	0.55	4	37	6	41
**chr10-69724940**	*Cct6b*	Intron	0.57	0.55	3	18	3	22
**chr20-11615350**	*Dnah8*	Exon	0.48	0.55	5	32	4	26
**chr1-197188202**	*Thumpd1*	Intron	0.55	0.55	7	59	6	43
**chr2-19664251**	*Atp6ap1l*	Intron	-0.59	0.55	5	41	3	23
**chr14-46826974**	*AABR06078903.1*	Intron	0.64	0.56	7	94	10	137
**chr15-87845778**	*Lmo7*	Intron	-0.76	0.56	3	19	3	16
**chr16-71635177**	*Adam32*	Intron	0.50	0.56	8	99	9	143

**chr16-82786462**	*Cars2*	Intron	0.46	0.56	3	26	3	23
**chr6-102053463**	*Pygl*	Exon	-0.53	0.56	3	21	6	47
**chrX-109570343**	*Il1rapl2*	Intron	-0.57	0.56	3	22	3	20
**chr3-120905161**	*Slc28a2*	Exon	0.66	0.57	3	25	3	19
**chr2-2982624**	*Ttc37*	Intron	-0.53	0.57	4	30	4	26
**chr4-227505829**	*Clec2dl1*	Intron	-0.46	0.57	3	18	4	22
**chr8-95520468**	*Snx14*	Intron	0.54	0.57	4	30	5	34
**chr5-124354955**	*Dnajc6*	Intron	0.74	0.57	3	21	5	36
**chr15-87845769**	*Lmo7*	Intron	0.72	0.57	3	19	4	23
**chr13-26973829**	*Cntnap5b*	Intron	0.67	0.57	4	21	5	32
**chrX-128270884**	*Thoc2*	Intron	-0.50	0.57	3	17	3	16
**chr19-38796055**	*LOC100909409*	Intron	0.48	0.57	3	25	5	47
**chr17-91096563**	*LOC688970*	Intron	-0.77	0.57	3	24	4	23
**chr7-114498195**	*Ptk2*	Exon	-0.62	0.58	3	19	3	18
**chr3-171859478**	*Zfp64*	Intron	0.61	0.58	4	33	4	32

**chr1-183410818**	*Galnt18*	Intron	-0.49	0.58	3	24	3	18
**chr8-129643656**	*Ccdc13*	Intron	-0.68	0.59	3	19	3	20
**chr18-17222153**	*RGD1562608*	Intron	0.74	0.59	5	36	6	63
**chr14-37654754**	*Slain2*	Intron	0.52	0.59	3	21	6	43
**chr1-126954895**	*Fam189a1*	Intron	0.80	0.59	4	21	4	23
**chr19-10912214**	*Nlrc5*	Intron	0.70	0.59	3	19	3	18
**chr8-129068049**	*Ulk4*	Intron	-0.53	0.59	3	26	6	37
**chr20-28596316**	*Ctnna3*	Intron	0.46	0.59	6	50	8	49
**chr9-7677772**	*AABR06058265.2*	Exon	0.74	0.59	4	31	3	15
**chr19-319769**	*AABR06096719.1*	Intron	0.57	0.60	4	35	6	65
**chr4-42377107**	*Cftr*	Intron	-0.66	0.60	5	34	3	22
**chr9-11429089**	*AABR06058610.1*	Intron	-0.90	0.60	3	19	3	16
**chr10-71254068**	*Synrg*	Intron	-0.84	0.61	3	16	4	23
**chr19-337695**	*LOC100912892*	Intron	-0.45	0.61	3	21	6	47
**chr7-58219846**	*Tbc1d15*	Intron	-0.47	0.61	6	36	4	27
**chr19-37497022**	*LOC100363633*	Intron	0.51	0.61	3	23	4	28

**chr20-34929288**	*Ros1*	Intron	0.59	0.61	4	24	3	22
**chr4-141196975**	*LOC100364190*	Intron	0.46	0.61	3	19	3	18
**chr4-194906178**	*Mitf*	Intron	-0.45	0.61	4	24	3	24
**chr3-9136394**	*Gpsm1*	Exon	0.71	0.62	5	32	3	18
**chr1-75575066**	*Rdh13*	Intron	0.56	0.62	6	42	5	33
**chr9-113449818**	*Wash*	Intron	0.85	0.62	3	26	5	26
**chr4-203144438**	*Cntn4*	Intron	-0.66	0.62	3	19	3	19
**chr2-248719916**	*Ndst4*	Intron	0.57	0.62	4	26	5	33
**chr9-68365125**	*Pard3b*	Intron	-0.47	0.63	5	42	6	56
**chr12-13251697**	*Zfp394*	Intron	-0.72	0.63	4	24	4	25
**chr8-48574628**	*Dscaml1*	Intron	0.54	0.63	3	19	3	21
**chr19-37410939**	*LOC100363633*	ExonIntron	0.63	0.63	3	15	5	39
**chr7-100398611**	*Nsmce2*	Intron	-0.71	0.63	3	19	3	19
**chr14-46703935**	*LOC257642*	Intron	-0.60	0.63	3	16	4	21
**chr17-48687945**	*Sugct*	Intron	0.77	0.63	3	19	5	47

**chr19-38818544**	*LOC100359783*	Intron	0.62	0.64	4	28	6	55
**chr8-96092255**	*Mthfs*	Intron	0.75	0.64	3	23	3	17
**chr7-20634311**	*LOC300308*	Intron	0.86	0.64	3	25	3	18
**chr20-46739691**	*RGD1304770*	Exon	0.86	0.64	4	23	3	19
**chr1-229478633**	*Naa40*	Intron	0.62	0.64	3	16	4	25
**chr16-18689028**	*F2rl3*	Exon	-0.79	0.64	3	23	3	18
**chr3-12609876**	*Tsc1*	Intron	0.48	0.64	3	22	3	21
**chr12-22079253**	*RGD1559588*	Intron	0.47	0.64	9	114	10	98
**chr11-60567404**	*Phldb2*	Exon	0.74	0.64	4	21	3	22
**chr19-37441330**	*LOC100363633*	Intron	0.62	0.64	3	26	3	20
**chr20-6997061**	*RT1-CE4*	Exon	0.86	0.65	4	24	3	16
**chr7-2202388**	*AABR06047141.1*	Promoter	-0.45	0.65	4	31	5	38
**chr13-73019090**	*Hmcn1*	Intron	-0.56	0.65	3	17	3	25
**chr4-194826564**	*Mitf*	Intron	-0.58	0.65	3	20	3	16
**chr10-3068574**	*Snx29*	Intron	-0.69	0.65	3	22	3	23
**chr13-83322498**	*Rabgap1l*	Intron	-0.66	0.65	5	38	4	23

**chr19-394712**	*LOC100912892*	Intron	-0.56	0.66	3	20	4	32
**chrX-115144181**	*Tuba1a*	Exon	-0.73	0.66	4	29	3	21
**chrX-43866977**	*Acot9*	Intron	-0.51	0.66	6	48	6	60
**chr3-89423452**	*Ext2*	Intron	0.54	0.66	3	17	3	18
**chr9-42560551**	*Kansl3*	Intron	-0.61	0.66	3	26	3	20
**chr2-92257304**	*Cdh12*	Intron	-0.50	0.66	4	24	3	24
**chr1-142146520**	*Polg*	Exon	-0.46	0.66	3	19	4	25
**chr9-109781645**	*Efna5*	Intron	-0.48	0.67	4	23	3	19
**chr1-169323994**	*Capn5*	Intron	-0.45	0.67	3	21	3	17
**chrX-2099486**	*Uxt*	Intron	0.52	0.67	3	16	4	28
**chr16-19547128**	*Myo9b*	Exon	0.91	0.67	3	15	4	22
**chr1-183410821**	*Galnt18*	Intron	-0.63	0.67	4	27	4	24
**chr2-143772791**	*Spata5*	Intron	0.54	0.67	4	26	5	36
**chr6-134724578**	*Tc2n*	Intron	-0.50	0.67	3	21	5	34
**chr19-302178**	*AABR06096719.1*	Intron	0.61	0.67	4	30	3	21
**chr15-22933819**	*AABR06082338.1*	Exon	0.96	0.68	3	22	3	19

**chr12-6464453**	*Vom2r60*	Intron	-0.52	0.68	6	33	5	33
**chrX-111196279**	*Col4a6*	Intron	-0.62	0.68	7	50	6	46
**chr3-73785883**	*Pde1a*	Intron	-0.45	0.69	7	66	8	79
**chr3-168661253**	*Zmynd8*	Intron	0.58	0.69	5	32	4	26
**chr13-12719025**	*Cntnap5c*	Intron	-0.49	0.69	4	24	5	32
**chr7-20624714**	*LOC300308*	Intron	0.50	0.69	4	32	7	54
**chr2-202424566**	*LOC679811*	Intron	0.90	0.69	3	19	3	23
**chr3-153443231**	*Sdcbp2*	Intron	0.50	0.70	3	21	4	28
**chr16-6799238**	*Sfmbt1*	Intron	0.69	0.70	5	37	4	27
**chr2-164724527**	*Nbea*	Intron	-0.47	0.70	4	38	3	26
**chr4-194906373**	*Mitf*	Intron	-0.48	0.70	4	36	4	28
**chr1-106784302**	*Nell1*	Intron	-0.56	0.70	4	27	4	21
**chr4-32401721**	*Acn9*	Intron	-0.62	0.70	3	17	3	17
**chr20-51108194**	*Prdm1*	Intron	0.61	0.70	3	17	3	20
**chr15-58235235**	*Fndc3a*	Intron	-0.65	0.70	4	30	4	27
**chr1-259283629**	*Rnls*	Intron	0.52	0.71	3	31	4	35

**chr13-12618733**	*Cntnap5c*	Intron	-0.88	0.71	4	31	3	23
**chr7-20632305**	*LOC300308*	Intron	0.71	0.71	4	38	4	32
**chr15-9579569**	*LOC100364581*	ExonIntron	0.53	0.71	3	34	4	32
**chr19-68301773**	*Trim67*	Exon	0.76	0.72	3	23	3	16
**chr2-256745184**	*Gstcd*	Intron	0.60	0.72	4	26	3	15
**chr1-26345148**	*LOC100361380*	Intron	0.45	0.72	5	56	8	112
**chrX-57116745**	*Il1rapl1*	Intron	-0.59	0.73	4	29	4	27
**chr9-26325981**	*LOC501110*	Intron	0.52	0.73	5	36	5	43
**chr2-202533419**	*Mnd1*	Exon	0.72	0.74	4	29	4	23
**chr5-81257339**	*Snx30*	Intron	-0.70	0.75	3	17	3	22
**chr19-37516212**	*LOC100363633*	Intron	0.57	0.75	4	31	3	27
**chr13-82297368**	*Rn50_13_0822.1*	Intron	0.68	0.76	3	21	3	18
**chr14-46822996**	*AABR06078903.1*	Intron	-0.49	0.76	3	20	3	22
**chr7-71967610**	*Cpq*	Intron	0.66	0.77	3	22	3	18
**chr11-89876930**	*Arvcf*	Intron	0.50	0.77	3	18	3	18

**chr17-62024620**	*Mpp7*	Intron	-0.55	0.77	5	33	4	31
**chr1-26352163**	*LOC100361380*	Intron	0.53	0.77	4	27	3	24
**chr7-74022390**	*Stk3*	Intron	-0.59	0.77	3	19	3	17
**chr2-191651454**	*Schip1*	Intron	0.57	0.77	6	47	7	43
**chr15-87160695**	*Pibf1*	Intron	0.66	0.78	3	18	3	22
**chr12-1491156**	*5S_rRNA*	Exon	0.45	0.78	3	18	3	27
**chr14-100519428**	*Egfr*	Intron	0.51	0.78	3	18	4	28
**chr19-39874540**	*AABR06098126.1*	Intron	0.62	0.79	4	29	4	24
**chr15-113593705**	*Itgbl1*	Intron	0.69	0.79	4	25	4	24
**chr7-58219870**	*Tbc1d15*	Intron	-0.82	0.79	3	19	4	25
**chr2-71024181**	*AABR06013141.2*	Exon	-0.56	0.79	3	27	5	28
**chr4-151173202**	*Pde1c*	Intron	-0.48	0.80	5	34	4	30
**chr17-28946208**	*Bmp6*	Intron	0.84	0.80	3	27	3	18
**chr13-39644388**	*Clasp1*	Intron	0.56	0.80	3	17	6	33
**chr19-37448800**	*LOC100363633*	Intron	0.51	0.82	3	21	4	22

**chr1-204961612**	*Eif3c*	Promoter	-0.54	0.83	4	36	5	36
**chr2-226808985**	*St7l*	Intron	-0.46	0.83	3	21	4	20
**chr14-46200048**	*LOC364157*	Intron	0.66	0.83	3	18	3	19
**chr13-54434209**	*Nfasc*	ExonIntron	-0.45	0.83	5	38	3	20
**chr9-1380927**	*Tbc1d5*	Intron	0.53	0.83	3	18	3	17
**chr3-168661232**	*Zmynd8*	Intron	0.68	0.83	5	31	4	27
**chr14-113859908**	*Eml6*	Intron	0.61	0.84	3	29	3	24
**chr16-77032746**	*AABR06089318.1*	Intron	0.65	0.85	3	16	3	19
**chr2-216013971**	*LOC100911180*	Exon	-0.78	0.86	3	18	3	17
**chr7-2197740**	*AABR06047140.1*	Exon	0.58	0.86	4	35	6	35
**chr6-111985569**	*LOC100909961*	Promoter	0.48	0.86	3	20	3	22
**chr8-24100540**	*Bbs9*	Intron	0.77	0.86	3	20	3	15
**chr7-78223365**	*Dcaf13*	Intron	-0.74	0.88	3	18	3	18
**chr1-54699127**	*Rps6ka2*	Intron	0.64	0.88	3	22	4	22
**chr14-46701845**	*LOC257642*	Intron	0.51	0.89	4	29	4	42
**chr4-154067628**	*Fam13a*	Intron	-0.46	0.90	3	29	3	29

**chr1-26351817**	*LOC100361380*	Intron	0.70	0.92	3	16	3	21
**chr14-46701852**	*LOC257642*	Intron	0.56	0.92	4	33	4	44
**chr17-44044765**	*RGD1307443*	Intron	0.70	0.92	3	19	3	16
**chr17-48688336**	*Sugct*	Intron	-0.61	0.93	3	16	3	17
**chr4-189491314**	*Ccdc174*	Intron	-0.64	0.94	4	28	3	22
**chr19-38815594**	*LOC100359783*	Exon	0.55	0.94	3	24	4	32
