# Supplementary material for: DNA Methylation of Synaptic Genes in the Prefrontal Cortex Is Associated with Aging and Age-Related Cognitive Impairment
Source: Front Aging Neurosci. 2017 Aug 2;9:249. doi: 10.3389/fnagi.2017.00249 (PMC5539085; doi:10.3389/fnagi.2017.00249)
Supplement: Supplementary file 6 [file Table_6.PDF]

Supplementary Table 6. Promoter and Gene Body features in CG and non-CG context in the mPFC in Young and Aged

	Young CG	Aged CG	Young CHG	Aged CHG	Young CHH	Aged CHH
**Average depth**	63.8	130.7	58.4	127.0	55.5	122.4
**Sites in young and aged**	16090	16090	51099	51099	128268	128268
**Mean methylation ratio**	0.731	0.730	0.023	0.023	0.018	0.018
**Sites ≤ 0.1**	557 (3.5%)	573 (3.6%)	48877 (95.7%)	49219 (96.3%)	123719 (96.5%)	124185 (96.8%)
