# Supplementary material for: DNA Methylation of Synaptic Genes in the Prefrontal Cortex Is Associated with Aging and Age-Related Cognitive Impairment
Source: Front Aging Neurosci. 2017 Aug 2;9:249. doi: 10.3389/fnagi.2017.00249 (PMC5539085; doi:10.3389/fnagi.2017.00249)
Supplement: Supplementary file 7 [file Table_7.PDF]

Supplementary Table 7. Promoter and Gene Body features in CG and non-CG context in the mPFC in AI and AU

	AU CG	AI CG	AU CHG	AI CHG	AU CHH	AI CHH
**Average depth**	77.6	67.3	74.3	62.4	71.2	59.2
**Sites in AU and AI**	14696	14696	48524	48524	123892	123892
**Mean methylation ratio**	0.722	0.723	0.023	0.023	0.018	0.018
**Sites ≤ 0.1**	583 (4.0%)	553 (3.8%)	46543 (95.9%)	46349 (95.5%)	119699 (96.6%)	119465 (96.4%)
