# Supplementary material for: DNA Methylation of Synaptic Genes in the Prefrontal Cortex Is Associated with Aging and Age-Related Cognitive Impairment
Source: Front Aging Neurosci. 2017 Aug 2;9:249. doi: 10.3389/fnagi.2017.00249 (PMC5539085; doi:10.3389/fnagi.2017.00249)
Supplement: Supplementary file 8 [file Table_8.PDF]

Supplementary Table 8. CHG hypermethylation of genes within GO clusters in animals with delayed shifting

Gene Symbol	Gene Name	Synapse	Kinase activity	GTPase activity
Dennd1a	DENN domain containing 1A	X		X
Erc2	ELKS/RAB6-interacting/CAST family member 2	X		
Fchsd2	FCH and double SH3 domains 2	X		
Fyn	FYN proto-oncogene, Src family tyrosine kinase	X	X	
Amph	amphiphysin	X		X
Ank3	ankyrin 3	X		
Ctnna2	catenin alpha 2	X		
Dgki	diacylglycerol kinase, iota	X	X	X
Dbi	diazepam binding inhibitor, acyl-CoA binding protein	X		
Erbb4	erb-b2 receptor tyrosine kinase 4	X	X	
Exoc4	exocyst complex component 4	X		
Gria4	glutamate ionotropic receptor AMPA type subunit 4	X		
Igf2bp1	insulin-like growth factor 2 mRNA binding protein 1	X		
Ica1	islet cell autoantigen 1	X		
Mapk10	mitogen activated protein kinase 10	X	X	
Musk	muscle associated receptor tyrosine kinase	X	X	
Kcnd2	potassium voltage-gated channel subfamily D member 2	X		
Ptprn2	protein tyrosine phosphatase, receptor type N2	X		X
Pcdh15	protocadherin 15	X		
Rims2	regulating synaptic membrane exocytosis 2	X		
Sorcs3	sortilin-related VPS10 domain containing receptor 3	X		
Sv2b	synaptic vesicle glycoprotein 2b	X		
Unc5c	unc-5 netrin receptor C	X		
Akap6	A-kinase anchoring protein 6		X	
Epha10	EPH receptor A10		X	
Epha3	Eph receptor A3		X	X
Fggy	FGGY carbohydrate kinase domain containing		X	
Tnni3k	TNNI3 interacting kinase		X	

Tnik	TRAF2 and NCK interacting kinase	X	
Mast4	microtubule associated serine/threonine kinase family member 4	X	
Map3k4	mitogen activated protein kinase kinase kinase 4	X	
Myo3a	myosin IIIA	X	
Obscn	obscurin, cytoskeletal calmodulin and titin-interacting RhoGEF	X	
Pi4ka	phosphatidylinositol 4-kinase alpha	X	
Prkg1	protein kinase, cGMP-dependent, type 1	X	X
Ptk2	protein tyrosine kinase 2	X	
Rps6ka6	ribosomal protein S6 kinase A6	X	
Stk39	serine threonine kinase 39	X	
LOC300308	similar to hypothetical protein 4930509O22	X	
LOC688970	similar to serine/threonine kinase	X	
Garnl3	GTPase activating Rap/RanGAP domain-like 3		X
Rab3gap2	RAB3 GTPase activating non-catalytic protein subunit 2		X
Rasgrf1	RAS protein-specific guanine nucleotide-releasing factor 1		X
Ralgps2	Ral GEF with PH domain and SH3 binding motif 2		X
Sbf2	SET binding factor 2		X
Srgap3	SLIT-ROBO Rho GTPase activating protein 3		X
Tbc1d4	TBC1 domain family, member 4		X
Tbc1d5	TBC1 domain family, member 5		X
Dock9	dedicator of cytokinesis 9		X
Elmo1	engulfment and cell motility 1		X
Obscn	obscurin, cytoskeletal calmodulin and titin-interacting RhoGEF		X
LOC691033	similar to GTPase activating protein testicular GAP1		X
LOC304239	similar to RalA binding protein 1		X
